# KAI1(CD82) is a key molecule to control angiogenesis and switch angiogenic milieu to quiescent state

**DOI:** 10.1186/s13045-021-01147-6

**Published:** 2021-09-16

**Authors:** Jin-Woo Lee, Jin Hur, Yoo-Wook Kwon, Cheong-Whan Chae, Jae-Il Choi, Injoo Hwang, Ji-Yeon Yun, Jin-A Kang, Young-Eun Choi, Young Hyun Kim, Sang Eun Lee, Cheol Lee, Dong Hyun Jo, Heeyoung Seok, Byong Seung Cho, Sung Hee Baek, Hyo-Soo Kim

**Affiliations:** 1 National Research Laboratory for Stem Cell Niche, Center for Medical Innovation, Seoul National University Hospital, Seoul, Republic of Korea; 2 Center of Cell- and Bio-Therapy (CBT), Seoul National University Hospital, Seoul, Republic of Korea; 3 Department of Convergence Medicine, Pusan National University School of Medicine, Yangsan, Republic of Korea; 4 Department of Pathology, Seoul National University College of Medicine, Seoul, Republic of Korea; 5 Department of Anatomy, Seoul National University College of Medicine, Seoul, Republic of Korea; 6 Creative Research Initiative Center for Chromatin Dynamics, School of Biological Sciences, Seoul National University, Seoul, 151-742 Republic of Korea; 7 Department of Molecular Medicine and Biopharmaceutical Sciences, Graduate School of Convergence Science and Technology, and College of Medicine or College of Pharmacy, Seoul National University, Seoul, Republic of Korea; 8 Genomics Core Facility, Department of Transdisciplinary Research and Collaboration, Biomedical Research Institute, Seoul National University Hospital, Seoul, Republic of Korea; 9 ExoCoBio Inc, Gasan digital 1-ro, Geumcheon-gu, Seoul, 08594 Republic of Korea; 10 Department of Internal Medicine, Seoul National University Hospital, 101 Daehak-ro, Jongno-gu, Seoul, 110-744 Korea

**Keywords:** Anti-angiogenesis, Endogenous angiogenic growth factor inhibitor, KAI1 (CD82), Pericyte

## Abstract

**Background:**

Little is known about endogenous inhibitors of angiogenic growth factors. In this study, we identified a novel endogenous anti-angiogenic factor expressed in pericytes and clarified its underlying mechanism and clinical significance.

**Methods:**

Herein, we found Kai1 knockout mice showed significantly enhanced angiogenesis. Then, we investigated the anti-angiogenic roll of Kai1 in vitro and in vivo.

**Results:**

KAI1 was mainly expressed in pericytes rather than in endothelial cells. It localized at the membrane surface after palmitoylation by zDHHC4 enzyme and induced LIF through the Src/p53 pathway. LIF released from pericytes in turn suppressed angiogenic factors in endothelial cells as well as in pericytes themselves, leading to inhibition of angiogenesis. Interestingly, KAI1 had another mechanism to inhibit angiogenesis: It directly bound to VEGF and PDGF and inhibited activation of their receptors. In the two different in vivo cancer models, KAI1 supplementation significantly inhibited tumor angiogenesis and growth. A peptide derived from the large extracellular loop of KAI1 has been shown to have anti-angiogenic effects to block the progression of breast cancer and retinal neovascularization in vivo.

**Conclusions:**

KAI1 from PC is a novel molecular regulator that counterbalances the effect of angiogenic factors.

**Supplementary Information:**

The online version contains supplementary material available at 10.1186/s13045-021-01147-6.

## Background

Angiogenesis is a process of new blood vessel formation from the preexisting vasculature. This process is mainly controlled by the interactions between two vascular cell types, endothelial cells (ECs), and pericytes (PCs) [1]. Although the role of ECs in angiogenesis has been extensively studied, that of PCs remains unclear except for in-vessel stabilization and paracrine signalling [2]. Previously, we revealed that KAI1 is a hypoxia-responsible gene and expressed in the ischemic myocardium and hypoxic bone marrow stem cell niche [3]. KAI1/CD82, a transmembrane protein and a member of the tetraspanin superfamily, is an evolutionally conserved molecule expressed in various tissue types. First identified to be involved in the T cell activation process, KAI1 is typically considered as a suppressor of metastasis. Most studies of KAI1 examined its function in suppressing metastasis and angiogenesis mainly in cancer cells and endothelial cells [4]. Recently, we and others have reported that KAI1 regulates the cell cycle progression of the long-term repopulating hematopoietic stem cells (LT-HSCs) [5, 6] and muscle stem-progenitor cells [5, 6]. Thus, KAI1 has different roles in each cell and organ type. However, the specific role of KAI1 in perivascular cells/pericytes is unclear.

Angiogenesis is strictly controlled by maintaining a proper balance between pro- and anti-angiogenic factors [2]. Most anti-angiogenic drugs used for cancer or vascular proliferative conditions block vascular endothelial growth factor (VEGF) signaling [7]. The mechanism of angiogenesis caused by VEGF is well-known [8, 9], but little is known about endogenous VEGF inhibitors and their control mechanism of the anti-angiogenic process. To date, at least 27 endogenous inhibitors of angiogenesis have been identified and detected in the blood [10, 11]. Most originate from the fragments of large extracellular matrix and non-matrix-derived molecules; however, tetraspanin superfamily-derived endogenous inhibitors of angiogenesis have not been reported. Here, we found that KAI1 expressed in PCs is an important endogenous counter-regulator that inhibits angiogenesis driven by growth factors such as VEGF and PDGF and that the homeostasis of angiogenesis is controlled by paracrine interactions between PCs and ECs.

## Materials and methods

### Mice

Adult C57BL/6 mice (6–12 weeks, male or female) were purchased from The Jackson Laboratory (Bar Harbor, ME). All animal experiments were approved by the Institutional Animal Care and Use Committee (IACUC) at Seoul National University Hospital and complied with the National Research Council (NRC) ‘Guidelines for the Care and Use of Laboratory Animals’.

### Generation of *Kai1-EmGFP* mouse

*Kai1/Cd82-EmGFP (Kai1-GFP)* mice were generated by Macrogen, Inc., using the CRISPR system. The mice were interbred and maintained under pathogen-free conditions. Protocols were reviewed and approved by the Institutional Animal Care and Use Committees (IACUC). C57BL/6N female mice were treated with pregnant mare serum gonadotropin and human chorionic gonadotropin. After 48 h, the female mice were mated with C57BL/6N stud male mice. On the next day, the vaginal plug was checked in female mice, and the mice were killed to harvest the fertilized embryos. Single-guide RNA (gRNA1: TCATTCTGAAGACTACAGCAAGG, gRNA2: AGCAAGGTCCCCAAGTACTGAGG), Cas9 nuclease, and dsDonor were mixed and microinjected into one cell embryo. Microinjected embryos were incubated at 37 °C for 1–2 h. Fourteen to sixteen injected one cell staged embryo were transplanted into the oviducts of pseudopregnant recipient mice (ICR). After the founders were born, genotyping of tail samples was performed by PCR (F1: CCCTTGTTAGTCC CCTCCTC, R1: TTACTTGTACAGCTCGTCCA; R2: CCCACACCCCT AAGTTGTCA). PCR-positive samples were subjected to TA cloning and analyzed by sequencing.

### Cells

MS1 (mouse endothelial cell line) and B16 (mouse melanoma cell line) cells were cultured in DMEM high glucose (Thermo Fisher Scientific) supplemented with 10% fetal bovine serum (FBS; Thermo Fisher Scientific) and 1× antibiotics-antimycotics (Thermo Fisher Scientific). 10T1/2 was maintained in RPMI 1640 HEPES (Thermo Fisher Scientific) supplemented with 10% FBS (Thermo Fisher Scientific) and 1× antibiotics-antimycotics (Thermo Fisher Scientific). Mouse primary perivascular cells (PVCs) and mouse primary aorta endothelial cells (ECs) were harvested and expanded in DMEM high glucose supplemented with 20% FBS. Human umbilical vein endothelial cells (HUVEC; human primary endothelial cell) were obtained from Lonza (Basel, Switzerland) and cultured in EGM-2MV (Lonza). HUVEC were cultured in 1.5% gelatin-coated dishes. Human brain vessel pericyte (HBVP, catalog no. #1200; ScienCell) was grown on poly-l-lysine-coated dishes (15ul diluted in 10 mL of distilled water) and maintained in Pericyte Medium (catalog no. #0413; ScienCell).

### Immunofluorescence analysis

Eyeballs including the optic nerve were collected from the mice at postnatal day 2 and 5. The retinae were stained with Lectin from Bandeiraea simplicifolia fluorescein isothiocyanate (FITC) conjugate (catalog no. L9381; Sigma-Aldrich), anti-CD31 phycoerythrin (PE) conjugate (catalog no. 340297; BD Biosciences), anti-NG2 Alexa 488 conjugate (catalog no. AB5320A4; Merck Millipore), anti-NG2 cy3 conjugate (catalog no. AB5320C3; Merck Millipore), anti-collagen IV antibody (catalog no. AB748; Merck Millipore), and anti-GFP (catalog no. A6455; Thermo Fisher Scientific). MS1 and 10T1/2 cells were fixed in 2% cold paraformaldehyde (Wako) for 10 min on ice. After washing with phosphate-buffered saline (PBS), the samples were blocked with 1% bovine serum albumin and 0.1% Triton X-100, followed by staining with anti-KAI1 (catalog no. SC-1087; Santa Cruz), anti-CD9 (catalog no. SC-13118; Santa Cruz), anti-alpha-smooth muscle actin Cy3 conjugate (catalog no. C6198; Sigma-Aldrich), anti-CD31 PE conjugate (BD Biosciences), and Bandeiraea simplicifolia fluorescein isothiocyanate conjugate (Sigma-Aldrich). After staining, the tissue and cells were examined by confocal microscopy (Zeiss).

### Gene expression analysis

Total RNAs were separated and purified and cell harvesting at representative time points. RNeasy® mini kit (catalog no. 74104; QIAGEN) and QIAshredder (catalog no. 79654; QIAGEN) were used to separate and purify RNA from cells. To synthesis the cDNA from RNA, we used qPCR RT master mix from Toyobo (catalog no. FSQ-201; TOYOBO). Real-time PCR was performed with SyBR Green I Mastermix (Applied Biosystems) using an ABI PRISM TM 7500 Sequence Detection System (Applied Biosystems). RT-PCR was performed using the S1000 Thermal Cycler (BIO-RAD) and detected the agarose gel with Gel Doc XR + Gel Documentation System (BIO-RAD). Information on the primers used is provided in the Supplemental Materials.

### Western blot

Cells were lysed with lysis buffer (catalog no. #9803s; Cell Signaling Technology) containing protease inhibitor cocktail (catalog no. #3100-001; Gendepot). Total protein was immunoblotted with primary antibodies against KAI1 (catalog no. ab135779; Abcam), LIF (catalog no. AB-449-NA; R&D Systems), tSrc, pSrc (Tyr^416^), and pSrc (Tyr^527^) (Cell Signaling Technology), Myc (catalog no. 05-724; Sigma-Aldrich), p53 (catalog no. MABE327; Merck Millipore), Pbx1 (catalog no. sc-889; Santa Cruz), Cav-1 (Caveolin-1, catalog no. sc-894; Santa Cruz), Flot1 (Flotillin1, catalog no. #3253s; Cell signaling Technology), p-VEGFR2 (catalog no. ab38473; Abcam), total VEGFR2 (catalog no. #2479s; Cell Signaling Technology), p-PDGFRβ (catalog no. #3166s; Cell signaling Technology), total PDGFRβ (catalog no. #4564s; Cell signaling Technology), PDGF-BB (catolg no. 07-1437; Sigma-Aldrich), and VEGF-A (catalog no. #ABS82; Merck Millipore) followed by incubation in horseradish peroxidase-conjugated secondary antibodies (Jackson Laboratory). GAPDH (catalog no. ab9485; Abcam) and beta-actin (catalog no. ab8227; Abcam) were used as an internal control.

### Flow cytometry

Cells were collected in FACS buffer [dPBS (Thermo Fisher Scientific) containing 1% FBS and 0.1% bovine serum albumin (Amresco)]. Cells were stained with antibodies, anti-CD82 (Miltenyi Biotec.), CD31 (Santa Cruz), and PDGFRβ (R&D Systems) for 30 min on ice washed out with FACS buffer after staining and analyzed with a BD FACSCanto II™ (BD Biosciences), followed by sorting using a BD FACSAria™ (BD Biosciences) instrument.

### Virus transduction

Adenovirus carrying mouse KAI1 was created with the pAdEasy vector system and titrated using the plaque-forming unit assay. 10T1/2 and MS-1 cells were transduced at a multiplicity of infection of 500 and 1000 pfu (plaque-forming unit), respectively, with 1 μg/mL polybrene (Sigma-Aldrich). For lentivirus transduction, MS1 cells were transduced by GFP encoded lentivirus using polybrene.

### In vitro angiogenesis assay

For the Matrigel two-dimensional tube formation assay, confocal dishes (Ibidi) were coated with 80 μL matrigel (Corning) and incubated for 30 min in a 37 °C incubator for polymerization. For EC-pericyte co-culture experiments, 3 × 10^4^ MS1 (EC) and 1 × 10^4^ WT/Kai1 K/O mouse primary PVCs were seeded onto the polymerized GFR-matrigel with EBM 5% media. For the EC-conditioned medium set, 3 × 10^4^ MS1 cells were seeded onto a matrigel coated µ-Dish (Ibidi) with conditioned media of primary PVCs from WT or Kai1 K/O mouse. For the three-dimensional spheroid sprouting assay, spheroids of PVCs, ECs, and B16 cells combinations were generated using the hanging drop method. Spheroids seeded onto polymerized matrigel. For the tube formation assay using an overconfluent PVC monolayer, a high density of 10T1/2 cells was incubated for 5 days in growth media and GFP expressing-ECs (MS1) were co-cultured with the PVC monolayer for 3 days.

Tube formation images were acquired by fluorescence microscopy and using a Zeiss LSM-710 META confocal microscope. Random fields were measured in terms of tube length and number of branching points by ImageJ software (National Institutes of Health).

### Lipid raft isolation

Lipid raft isolation in mouse PCs (10T1/2) and ECs (MS-1) was performed. For lipid-protein crosslinking, the cells were treated with 1.25 mM DTSSP (catalog no. 21578; Thermo Fisher Scientific) solution and incubated for 1 h on ice at 4 °C. The cells were washed with cold PBS and 5 mM EDTA and then 0.75 mL cold PBS and 5 mM EDTA were added. The samples were frozen overnight at − 80 °C. The cells were lysed with 0.8 mL 0.1% Triton X-100 membrane raft isolation buffer [1 M Tris–HCL, pH7.4, 1 M NaCl, 100 mM EDTA, Triton X-100, DW, protease inhibitor cocktail (500×)] by passing through a 23-gauge needle on a 5-mL syringe 20 times. Next, 500 μL supernatant 1 mL OptiPrep (catalog no. 1114542; Proteogenix) separation medium (60% iodixanol) were added, resulting in a 40% iodixanol solution of lysed cells. Using a Pasteur pipet, the 40% iodixanol solution was carefully overlaid with equal amounts of 30% iodixanol solution, followed by 5% iodixanol solution. The gradient was visible to the naked eye. Samples were ultra-centrifuged for 5 h at 132,000 g and 4 °C. The membrane rafts were present in the second fraction and were visible. Western blotting was performed as described above.

### Determining LIF concentrations in conditioned medium (ELISA)

Leukemia inhibitory factor (LIF) concentrations in the conditioned media of adenovirus-transduced MS1 and 10T1/2 cells were determined using a mouse LIF Quantikine ELISA Kit (R&D Systems). 1:2 diluted standard, control, and samples were incubated for 2 h at room temperature followed by a total of five washes. Next, 100 μL of mouse LIF Conjugate was added and incubated for 2 h at room temperature, followed by five washes, and then 100 μL of substrate solution was added and incubated for 30 min at room temperature in the dark. Finally, 100 μL of stop solution was added and the optical density of each sample was determined using a microplate reader (Promega). The concentration was calculated by four-parameter logistic (4-PL) regression.

### Acyl-biotin exchange (ABE) assay

mPVCs (101/2) and mECs (MS-1) were harvested by using palmitoylation lysis buffer (PLB) containing 1% NP-40, 10% glycerol, 50 mM *N*-ethylmaleimide, 1 μM PMSF (Sigma-Aldrich), 200X PIC (Biovision), 50 mM Tris–HCl pH 7.4, and 150 mM NaCl. Additionally, DHHC3 and DHHC4 knockdown mPVCs (101/2) by short interfering RNA (Santa Cruz) were harvested in PLB buffer. Lysates were briefly sonicated and incubated at 4 °C for 24 h, followed by centrifugation at 13,300 rpm 30 min at 4 °C. The supernatant was subject to chloroform–methanol protein precipitation (4:1:3 = methanol:chloroform:water). The protein pellet was solubilized in 0.5% SDS buffer and briefly sonicated. Concentrated proteins were treated with 0.5 M hydroxylamine (catalog no. 031-329-9000; Sigma-Aldrich) or Tris–HCl pH 7.4 buffer (negative control) for 2 h at room temperature. Next, the proteins were precipitated by the chloroform–methanol precipitation method, solubilized in 0.5% SDS, and then briefly sonicated. Solubilized proteins were diluted with 1/10 dilution to 0.05% SDS and immunoprecipitated with 40 μL neutravidin (Invitrogen) for 2 h at 4 °C. Immunoprecipitated beads were washed three times with PLB and eluted in 2× DTT-containing SDS-PAGE sample buffer (3 M). Samples obtained through the ABE assay were performed through the western blotting described above.

### RNA sequencing

RNA was extracted from pericytes of WT and *Kai1* K/O mice. RNA sequencing reads were aligned to the mouse genome build mm9 (NCBI37) using TopHat 2.0.9 and Bowtie 0.12.9 with a segment-length of 21, which allowed 2 mismatches per read. Expression levels for 23,170 RefSeq genes were measured by reads per kilobase per million mapped reads (RPKM). Differentially expressed genes between conditions were tested by Cuffdiff. Differentially expressed genes were defined as having more than a twofold change and an adjusted *p* value less than 0.05. Hierarchical clustering of samples was performed by R (www.R-project.org). Gene Ontology (GO) analysis was performed (http://geneontology.org/). Gene Set Enrichment Analysis (GSEA) was performed against custom-made lists from the GO database. The RNA-seq data have been deposited in the Gene Expression Omnibus (GEO) database (GSE114465).

### Chromatin immunoprecipitation (ChIP)

Putative p53 and pbx1 binding sites were predicted using the EpiTect ChIP qPCR Primers website (Qiagen, http://www.sabiosciences.com/chipqpcrsearch.php?app=TFBS&qs=1490097319). 10T1/2 cells were transfected with FLAG-tagged mouse KAI1 plasmid (KAI1 plasmid from R&D Systems, catalog no. RDC0331; pCMV-Tag 2B vector carrying FLAG obtained from Agilent Technologies) that had been incubated with polyethylenimine (Sigma-Aldrich) to improve transfection efficiency. For Src inhibition, 5 µM of PP2 (catalog no. P0042; Sigma-Aldrich) was added to the normal growth medium. The cells were fixed in 1% formaldehyde (catalog no. F8775; Sigma-Aldrich) for 10 min at room temperature for crosslinking and collected in a tube. The supernatant was discarded, and ChIP RIPA buffer (50 mM Tris–Cl, 150 mM NaCl, 10 mM Triton X-100, 1 mM EDTA, 0.1% sodium deoxycholate, 0.1% SDS, 1× protease inhibitor cocktail, 1 mM phenylmethane sulfonyl fluoride) was added to the pellet. To shear the DNA, both fractions were sonicated 10 times with a BIORUPTOR (30 s on and 30 s off per cycle; Diagenode). The immunoprecipitated sample was supplemented with a p53 (catalog no. MABE327; Merck Millipore) and pbx1 (catalog no. SC-889; Santa Cruz) antibody (2 µg) and rotated overnight. Protein A/G sepharose (catalog no. ab193262; Abcam) were added to pulldown the antibodies, and the samples were washed sequentially three times with homemade washing buffers 1, 2, and 3 (washing buffer 1: 20 mM Tris–Cl, 140 mM NaCl, 0.5 mM Triton X-100, 0.1 mM EDTA; washing buffer 2: 20 mM Tris–Cl, 500 mM NaCl, 0.5 mM Triton X-100, 0.1 mM EDTA; washing buffer 3: 20 mM Tris–Cl, 250 mM LiCl, 0.5 mM Triton X-100, 0.1 mM EDTA). Samples were incubated overnight at 65 °C for decrosslinking and recovered using a PCR purification kit (Qiagen). Recovered DNA was analyzed by semi-quantitative PCR.

### Luciferase assay

10T1/2 cells were transiently co-transfected with firefly pGL3-control luciferase reporter vector (Promega) and renilla luciferase reporter plasmid pRL-TK (catalog no. E2241; Promega) by using Neon transfection system (Thermo Fisher Scientific). Lif promoter binding affinity-luciferase activity was detected using Dual-Glo Luciferase Assay system (Promega), following the protocol provided by the manufacturer.

### Protein interaction assay

The surface plasmon resonance (SPR) binding studies were performed by Woojung BSC Inc. (Seoul, Korea) using a Biacore T200 (GE Healthcare, Sweden) instrument optical biosensor and CM5 chip (GE healthcare, Cat#. BR-1005-30, Sweden). The data were analyzed by using BIAevaluation software version 3.0 (GE Healthcare, Sweden). Real-time data showing the binding of recombinant KAI proteins to angiogenic cytokines was obtained using a Blitz instrument (ForteBio).

### Immunoprecipitation

Kai1-Gfp fusion vector and Dhhc-Ha vectors were co-transfected into 293T cells using ViaFect (Promega) according to the manufacturer’s instructions. pEF-Bos-zDHHC-HA constructs were a generous gift from Professor Masaki Fukata (National Institute for Physiological Science (NIPS). 293T cells were harvested in IP buffer (20 mM Tris–Cl, 140 mM NaCl, 10 mM NP-40, 10 mM Triton X-100, 0.5 mM EDTA, and 1× protease inhibitor cocktail) to confirm the interaction between Kai1-VEGF-A and Kai1-PDGF-BB, respectively, and incubated for 10 min at 4 °C. Following incubation, centrifuge for 10 min at 4 °C and lysates transfer to a new tube. Lysates were incubated for overnight at 4 °C with anti-VEGF-A (Merck Millipore) (or anti-PDGF-BB; Sigma-Aldrich) antibody or normal rabbit IgG (catalog no. sc-2027; Santa Cruz Biotechnology). After incubation with the antibody, the lysates were incubated with protein A/G sepharose (Abcam) for 2 h at 4 °C. After washing the beads were resuspended in 2× reducing sample buffer and heated for 5 min at 95 °C to dissociate captured antigen from beads. Samples obtained through the immunoprecipitation were performed through the western blotting described above.

### Duolink, proximity ligation assay (PLA)

10T1/2 cells were used for Kai1 and PDGF-BB, Kai1 and VEGF-A binding assay (PLA; Duolink Proximity Ligation). Cells were seeded on confocal dishes (Ibidi) and incubated for 24 h. After cells were attached, we treated 2-bp (5 μM) for 24 h. Then, cells were fixed 2% cold paraformaldehyde (Wako) for 10 min on ice and incubated with primary antibodies, Kai1 (catalog no. ab140238; Abcam), PDGF-BB (catolg no. 07-1437; Sigma-Aldrich), and VEGF-A (catalog no. #ABS82; Merck Millipore), as for IF. Duolink™ (catalog no. DUO92101; Sigma-Aldrich) experiments were performed using the protocol provided by Sigma-Aldrich. After PLA, the cells were examined by confocal microscopy (Zeiss).

### Mouse tumor graft model

For efficacy evaluation of rhKAI1 and KAI1 overexpressing cells, 2 × 10^6^ PC-3 (human prostate cancer cell line), B16 (mouse melanoma cancer cell line) cells and MDA-MB231 cells were mixed with rhKAI1 protein (catalog no. 12275-H08H; SinoBiological) 4 μg or conditioned media of vehicle or *Kai1* O/E 10T1/2 cell line or KAI1 peptide (wild-type sequence; NRPEVTYPCSCEVKGEEDNS, mutant sequence; NRPEVTAPASCEVKGAAANS), and subsequently embedded in 100 μL of matrigel (catalog no. 356231; Corning). NSG and C57BL/6 mice were subcutaneously injected with 100 μL of matrigel-cell mixture in the dorsal area. After 14 days, mice were killed in order to harvest tumor tissues. The tissues were fixed with 4% paraformaldehyde (Wako), paraffin-embedded and mounted on slide glasses. The samples were examined by immunofluorescence analysis.

### Oxygen-induced retinopathy (OIR) in mice

Newborn mice were exposed to hyperoxia (75 ± 0.5% O_2_) from postnatal day 7 to 12 and returned to normal room air. At postnatal day 14, we intravitreally injected 1 μL of PBS, KAI WT, rhKAI1 and KAI Mut into the right eyes of mice (*n* = 4). At P17, the enucleated eyes were prepared for immunofluorescent staining of whole-mount retinas with Alexa Fluor® 594 isolectin GS-IB4 conjugate (5 μg/mL; Invitrogen). The whole-mount retinas were viewed with a fluorescence microscope (Eclipse 90i; Nikon). Then, the neovascular tufts were marked and calculated using Image J (NIH). The area of neovascular tufts was normalized to the area of whole retina.

### Statistical analysis

All tests were repeated several times, independently, to verify reproducibility and the replicate number for each test is shown in each figure. We performed statistical analysis and used two-tailed Mann–Whitney rank sum tests using Prism software (version 5.00; GraphPad Software). The resulting *p* values are indicated as follows: NS, *p < 0.05, **p < 0.01, and ***p < 0.001. Data show the mean ± SEM.

## Results

### KAI1/CD82 shows anti-angiogenic effects

**Fig. 1:**
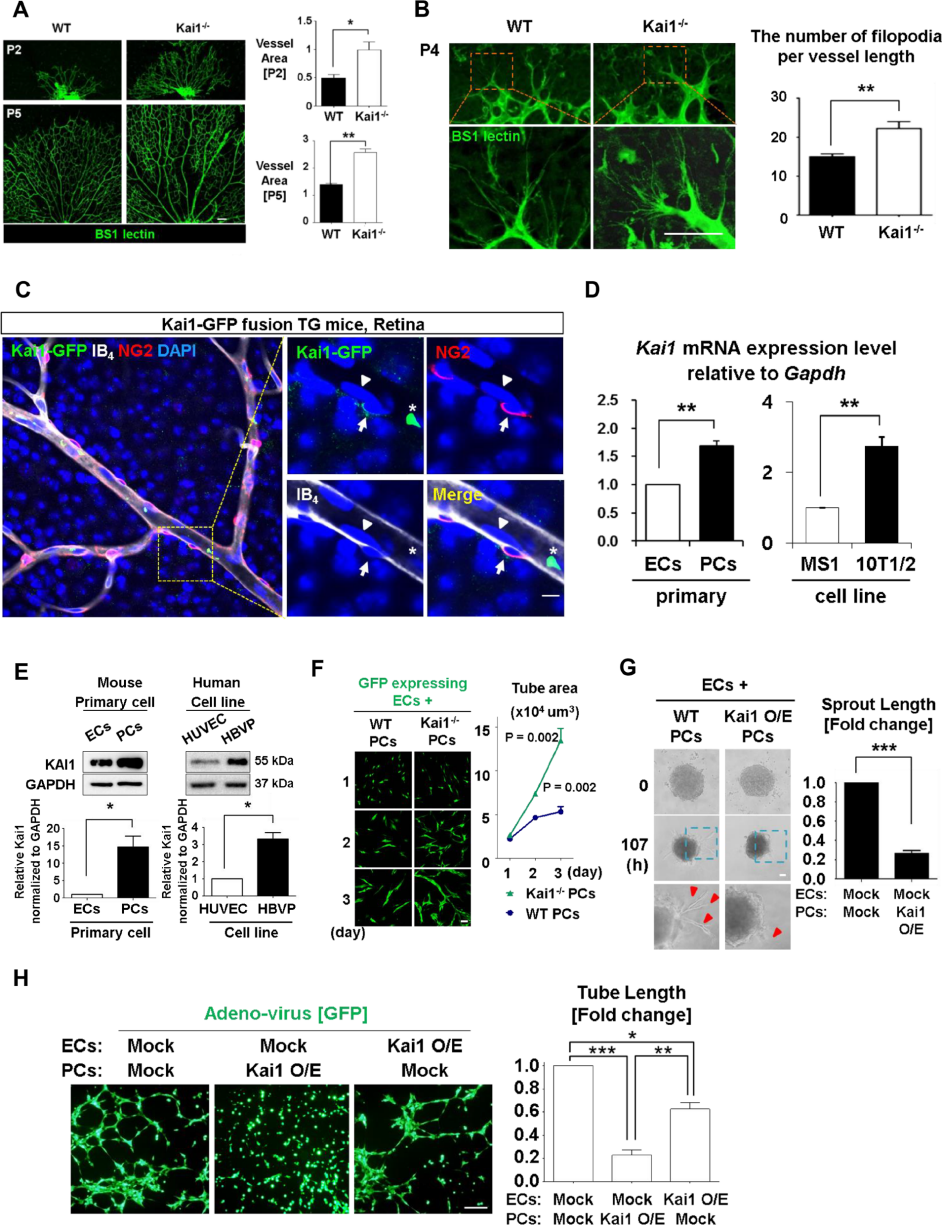
KAI1 was predominantly expressed in PCs rather than in ECs, and KAI1 expressed in PC has anti-angiogenic effects. **a** Left, retinal vasculature of the wild-type (WT) and Kai1 knockout (Kai1−/−) mice (postnatal day 2 and 5 days of age). Endothelial cells were stained with BS-I lectin. Scale bar, 100 μm. Right, quantification of the obtained results (Means ± SEM, 4–9 technical replicates). **b** Left, filopodia of retinal vessels in WT and Kai1−/− mice (postnatal day 4). Scale bar, 20 μm. Right, quantification of the obtained results (*p < 0.01, **p < 0.05, Means ± SEM, 3 or 4 technical replicates). **c** Tracking of Kai1 expressing cells in retina of the Kai1-GFP fusion knock-in mice. Confocal images of isolectin B4 (IB4, white), NG2 (red). Right, high-magnification images of the boxed area in the left panel Kai1 (green) was expressed in NG2 positive PCs (Arrow), but not ECs (Arrowhead). Asterisk indicates Kai1-expressing leukocyte or erythrocyte in vessel. Scale bar, 5 μm. **d** *Kai1* mRNA expression in mouse primary ECs and cell lines (MS1, endothelial cell line), and mouse primary PCs and cell line (10T1/2, pericyte cell line). **p < 0.05. **e** KAI1 protein expression in the mouse primary ECs, mouse primary PCs, human cell lines, HUVECs and HBVPs. *p < 0.01. **f** Left, in vitro co-culture matrigel tube formation assay using GFP expressing mouse ECs (MS1) co-cultured with *Kai1*^−/−^ or WT mouse primary PCs. Scale bar, 100 μm. MS1 cells were transduced by lentivirus encoding GFP. Right, quantification of the obtained results (Means ± SEM, 7 technical replicates). **g** Left, In vitro matrigel tube formation assay, performed using co-culture spheroids, consisting of primary ECs and PCs, transduced with Myc-Kai1 vector and observed using time-lapse microscopy at the indicated time, 0 and 107 h. Scale bar, 50 μm. Right, quantification of the obtained results (***p < 0.001, Means ± SEM, 3 technical replicates). **h** Left, in vitro tube formation assay in MS1 and 10T1/2 co-cultured cells, transduced with the indicated adenovirus. Scale bar, 200 μm. Right, quantification of the obtained results (*p < 0.01, **p < 0.05, ***p < 0.001, Means ± SEM, 4 technical replicates)

*Kai1* knockout (*Kai1*−/−) mice exhibited the accelerated retinal vascularization, as observed in the immunofluorescence (IF) images of the retinal vascular bed compared to in wild-type (WT) mice (Fig. 1a). Additionally, *Kai1*−/− retinae at postnatal 4 day displayed more filopodia (Fig. 1b) and fewer empty sleeves (i.e. less vessel regression) compared to in WT mice (Additional file 1: Fig. EV1A).

To determine whether the ECs or PCs were responsible for the enhanced angiogenesis in *Kai1*−/− mice, we analyzed Kai1-expressing cells in the vascular niche. To trace the Kai1 protein expression among various types of cells in vivo, we generated transgenic mice expressing the Kai1-GFP fusion protein using the CRISPR system. PCs and ECs of *Kai1-GFP* mice were identified by NG2 and IB4, respectively, located at the cell surface. Interestingly, KAI1 was predominantly expressed in PCs (NG2^+^IB4^−^) rather than in ECs (NG2^−^IB4^+^) of the *Kai1-GFP* mouse retinae (Fig. 1c). Similar results were obtained by immunofluorescent staining for KAI1, NG2, and CD31 in WT mice retinae (Additional file 1: Fig. EV1B). In the brain and heart, KAI1 was expressed in the PCs (Additional file 1: Fig. EV1C). Fluorescence-activated cell sorting (FACS) analysis revealed that 43% of PDGFRβ^+^ PCs were Kai1^+^ cells in the bone marrow of the *Kai1-GFP* fusion transgenic mice, in contrast, only 9% of ECs were Kai1 positive (Additional file 1: Fig. EV1D). Additionally, we analyzed the expression of this molecule in the mouse cell lines (ECs; MS1, PCs; 10T1/2) and mouse primary-cultured aortic ECs and PCs. PCs exhibited considerably higher Kai1 expression levels compared to ECs (Fig. 1d, e). Similar results were obtained by FACS analysis of HUVECs versus human brain vessel PCs (HBVPs) (Additional file 1: Fig. EV1E). Next, we evaluated the mRNA expression of *Kai1* and other tetraspanin superfamily members in PCs and ECs. *Cd9* and *Cd151* as well as *Cd82* (*Kai1)* were expressed predominantly in PCs (Additional file 1: Fig. EV1F and G).

To evaluate the effects of Kai1 in PCs on angiogenesis, we co-cultured *Kai1*−/− mice primary PCs with MS1 cells (mouse EC line) and found that the co-culture matrigel tube formation assay was significantly enhanced, compared to co-culture of WT mice primary PCs and MS1 cells (Fig. 1f). In contrast, Kai1-overexpressing PC suppressed ECs sprouting in the co-culture spheroid assay in mouse primary cells and cell lines (Fig. 1g, Additional file 1: Fig. EV1H and Additional file 1: movie EV1A, and B). To confirm the cell of origin of Kai1 for inhibiting angiogenesis, we prepared mock- or Kai1-O/E MS1 or 10T1/2 cells for a matrigel tube formation assay under various combinations of co-culture. We found that overexpression of Kai1 in the PCs led to strong suppression of tube formation, whereas overexpression in ECs showed a relatively weak effect (Fig. 1h).

### KAI1 localizes at the lipid rafts of PCs, which depends on palmitoylation

**Fig. 2:**
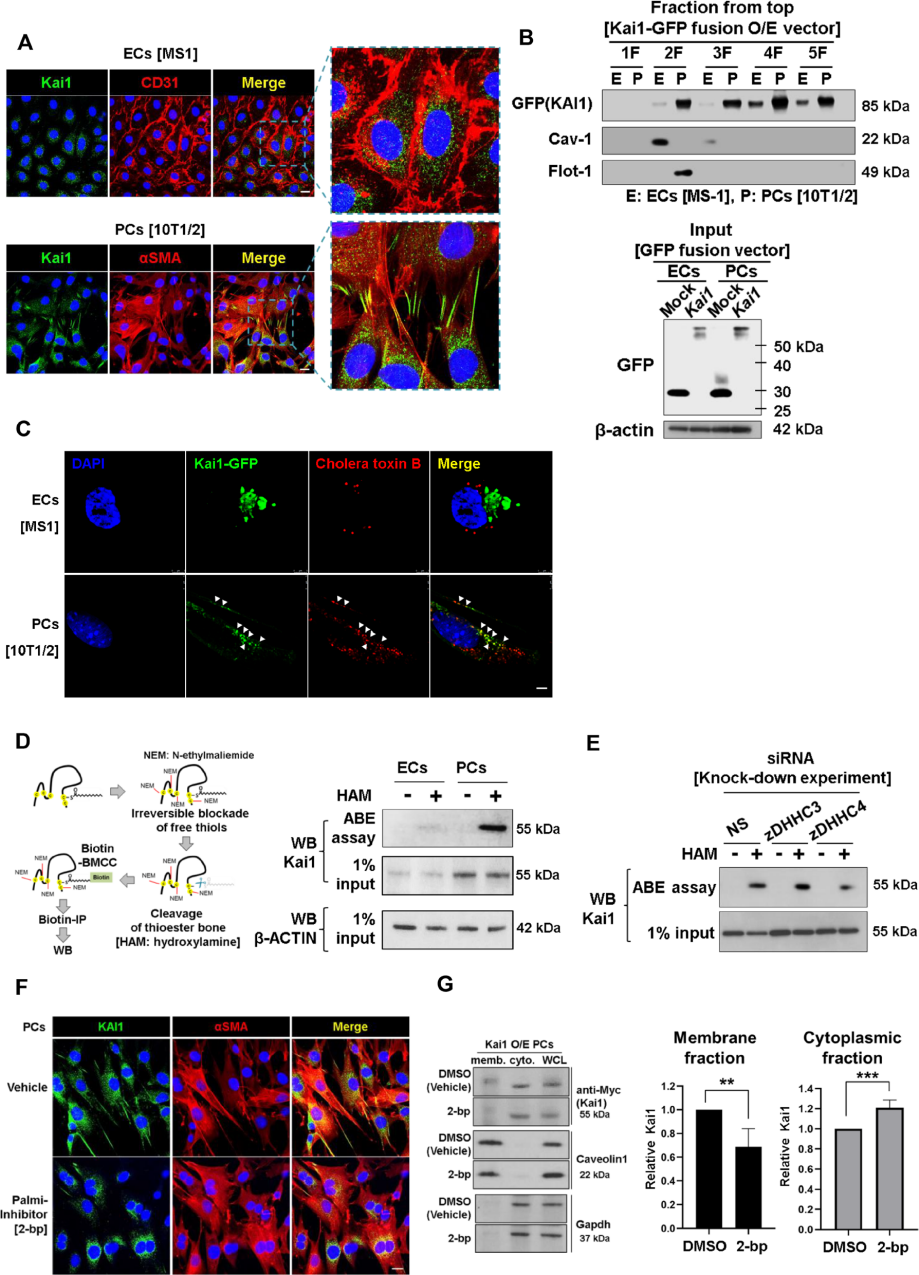
KAI1 localizes at the lipid rafts of PCs, which depends on palmitoylation. **a** Kai1 localization confirmed in MS1 and 10T1/2 cells using immunofluorescence. Scale bar, 50 μm. **b** Lipid rafts isolated from MS1 and 10T1/2 cells transfected with Kai1-GFP fusion vector using OptiPrep medium (frozen and crosslinks method). Flotillin-1 (planar type) and caveolin-1 (caveolae type) was detected in the lipid-raft membrane fraction. Fraction 1: no cellular protein. Fraction 2; Lipid raft proteins. Fraction 3; Non-raft membrane proteins. Fraction 4 and 5; Cytosolic proteins. Input samples are shown below. **c** Lipid raft marker (Cholera toxin B, red) co-localization with Kai1 (green) on MS1 and 10T1/2 cells transfected with Kai1-GFP fusion vector, observed using structured illumination microscopy. Arrowheads, co-localization of Kai1 protein and cholera toxin B. Scale bar, 2 μm. **d** Kai1 palmitoylation was analyzed by Acyl-Biotin Exchange (ABE) assay in ECs (MS1) and PCs (10T1/2). **e** zDHHC3 and zDHHC4 knockdown in 10T1/2, and their effects on Kai1 palmitoylation levels. **f** Changes in Kai1 localization after inhibition of palmitoylation using 2-bromopalmitate (2-bp) in 10T1/2. Scale bar, 50 μm. **g** Left, changes in Kai1 localization in the membrane and cytosol of 10T1/2 cells following the inhibition of palmitoylation using 2-bp. Right, quantification of the obtained results (**p < 0.05, ***p < 0.001, Means ± SEM, 3 technical replicates)

To understand the different effects of KAI1 in ECs and PCs, we analyzed the localization of KAI1. Interestingly, surface expression of Kai1 was much stronger on mouse PCs (10T1/2) than on mouse ECs (MS1) (Fig. 2a). Next, we performed subcellular fractionation to isolate the lipid raft membrane, non-lipid raft membrane, and cytosolic proteins in ECs and PCs. Endogenous Kai1 was detected in the membranous and cytosolic fractions of 10T1/2, but not detected in MS1 (Additional file 1: Fig. EV2A). When Kai1-GFP was introduced, both PCs and ECs expressed Kai1 in the cytosolic fraction. Interestingly, however, Kai1 expression in the membranous or lipid-raft fraction differed; expression was strong in 10T1/2 and very weak in MS1 (Fig. 2b). Expression of Kai1 in the lipid raft on 10T1/2 rather than on MS1 was confirmed by analyzing the expression of a lipid raft marker, cholera toxin B, and its co-localization with Kai1 on 10T1/2 (Fig. 2c). Two different types of the lipid rafts have been reported: flotillin-enriched planar lipid rafts or caveolin-enriched caveolae. We found that PCs had flotillin-enriched planar lipid rafts whereas ECs had the caveolae type (Fig. 2b).

Next, we examined the mechanism of Kai1 localization at the lipid raft membranous fraction of PCs, but not ECs. Palmitoylation is an important mechanism to regulate functions of the tetraspanin family members including CD9, CD81, KAI1/CD82, and CD151. KAI1 is palmitoylated at cytoplasmic cysteine residues, and then localizes to the cell membrane [12]. It is known that the functions of KAI1 depend on its palmitoylation status [12, 13]. Therefore, we hypothesized that the different distribution or effect of KAI1 between ECs and PCs were related to the different status of KAI1 palmitoylation between these cells. To detect the palmitoylated KAI1 molecules in ECs and PCs, we performed acyl-biotin exchange (ABE) assay. The palmitoylation level of Kai1 was significantly higher in PCs than in ECs (Fig. 2d). Protein palmitoylation is catalyzed by zDHHC palmitoyl transferases, which contain cysteine-rich domains (CRDs) [14]. Members of the zDHHC family, except for zDHHC12, showed higher expression in PCs than in ECs (Additional file 1: Fig. EV2B). We demonstrated that Kai1 directly interacts with zDHHC3 and zDHHC4 among the 23 zDHHC enzymes screened (Additional file 1: Fig. EV2C). To evaluate Kai1 palmitoylation by these two enzymes, we performed an ABE assay following the knockdown of these genes. Interestingly, Kai1 palmitoylation was reduced by zDHHC4 knockdown, but not by zDHHC3 knockdown (Fig. 2e). Inhibition of palmitoylation using 2-bromopalmitate (2-bp) significantly inhibited the Kai1 localization in the lipid raft or membrane of 10T1/2 (Fig. 2f, g).

### KAI1 induces LIF through the Src/p53 axis to downregulate angiogenic genes

**Fig. 3:**
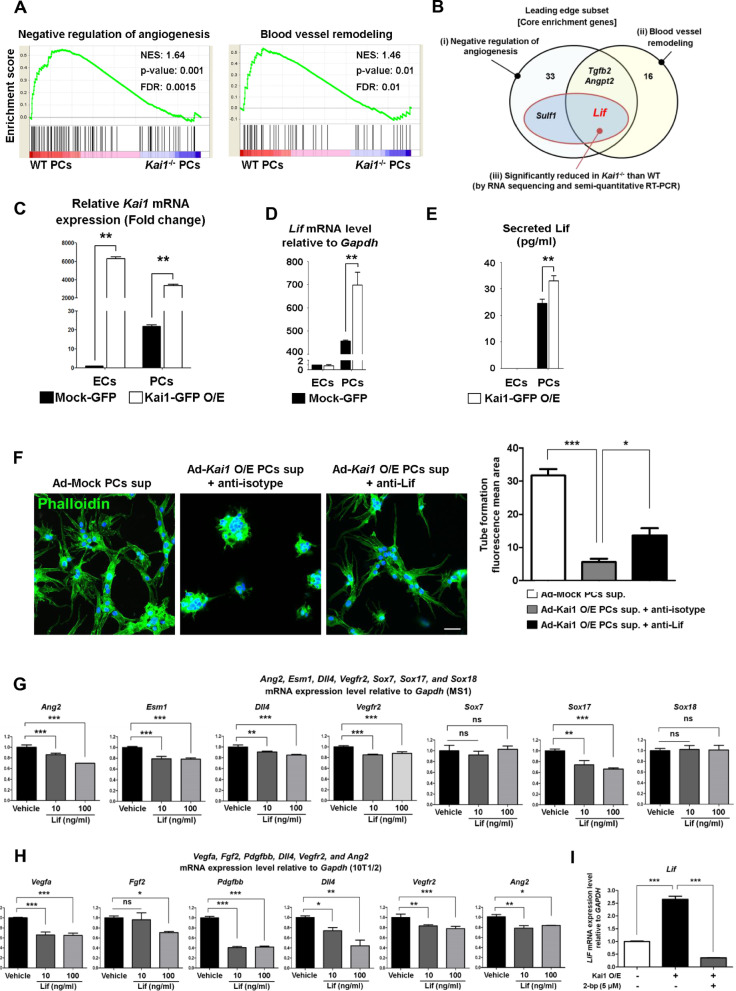
Anti-angiogenic LIF is the downstream effect molecule induced by KAI1. **a** GSEA of the “negative angiogenic regulators” (GO: 0016525) and “blood vessel remodelling” (GO: 0001974), based on the RNA sequencing of WT and *Kai1*^−/−^ PC. **b** Venn diagram showing the leading edge subset (core enrichment genes) of GSEA data for “negative angiogenic regulators” (GO: 0016525) and “blood vessel remodelling” (GO: 0001974), based on the RNA sequencing and semi-quantitative RT-PCR of WT and *Kai1*^−/−^ PC. **c** qRT-PCR analysis for *Kai1* mRNA level in Kai1-O/E ECs (MS1) and PCs (10T1/2). The genes were normalized by *Gapdh* (**p < 0.05, Means ± SEM, 3 technical replicates). **d** Lif expression in the Kai1-O/E MS1 and 10T1/2 cells (Means ± SEM, 3 technical replicates). **e** Lif level determination in the conditioned media of Kai1-O/E MS1 and 10T1/2 cells (Means ± SEM, 5 technical replicates). **f** Top, Tube formation assay using MS1 cells cultured in the conditioned media obtained from the mock and Kai1-O/E 10T1/2. These media were treated with control IgG or Lif antibody. Scale bar, 50 μm. Bottom, Quantification of the obtained results (*p < 0.01, **p < 0.05, ***p < 0.001, Means ± SEM, 3 technical replicates). **g** Changes in the levels of angiogenic molecules (*Ang2*, *Esm1*, *Dll4*, *Vegfr2*, *Sox7*, *Sox17*, and *Sox18*) by using semi-quantitative RT-PCR in MS1 cells following the treatment with mouse Lif (10 and 100 ng/mL). ns; not significant, **p < 0.05, ***p < 0.001. **h** Changes in *Vegfa*, *Fgf2*, *Pdgfbb*, *Vegfr2*, *Dll4* and *Ang2* levels in 10T1/2 cells in response to the Lif treatment (10 and 100 ng/mL). ns; not significant, *p < 0.01, **p < 0.05, ***p < 0.001. **i** After palmitoylation inhibition with 2-bp in 10T1/2 cells, *Lif* expression was detected by qRT-PCR. ***p < 0.001

To investigate the mechanisms by which KAI1 negatively regulates angiogenesis, we analyzed the transcriptome of WT and Kai1^−/−^ primary PCs and performed gene set enrichment analysis (GSEA). We found that Kai1^−/−^ PCs expressed significantly lower level of “negative angiogenic regulators” (GO: 0016525) and “blood vessel remodelling genes” (GO: 0001974) compared to control PCs did (Fig. 3a).

Among top 17 of “negative angiogenic regulators” (GO: 0016525), *Sulf1* and *Lif* levels were considerably decreased in *Kai1*−/− primary PCs to less than 20% of those in control PCs (Additional file 1: Fig. EV3A). Interestingly, Leukemia inhibitory factor (Lif) which is both a negative regulators of angiogenesis and the blood vessel remodelling genes (Fig. 3a, b) emerged as a member of the leading edge subset that contributing to the enrichment score (ES) [15]. A previous study reported that *Lif*−/− mice have increased endothelial filopodia, branching points, and capillary density in the retina [16], which is very similar to our findings in *Kai1*−/− mice. Thus, we examined *Lif* mRNA expression in PCs and ECs after Kai1 O/E (Fig. 3c), and found that Kai1 significantly increased the mRNA levels of *Lif* in PCs, but not in ECs (Fig. 3d). The Lif protein concentration in the conditioned medium obtained from Kai1-O/E PCs was shown to be higher than that in the medium obtained from control PCs (Fig. 3e). And G0 arrest was increased in the ECs treated with conditioned medium obtained from Kai1-O/E PCs (Additional file 1: Fig. EV3B).

The baseline expression level of Lif was also high in PCs compared to in ECs in mouse and human cells. However, the expression level of its receptor LIFR was comparable between PCs and ECs in both species, mouse and human (Additional file 1: Fig. EV3C). To examine the functional relevance of Lif during angiogenesis, we performed a tube formation assay after neutralization of Lif in the supernatant of Kai1-O/E PCs (10T1/2). Interestingly, the supernatant of Kai1-O/E PCs inhibited the tube formation of ECs (MS1), which was reversed by neutralization of Lif (Fig. 3f).

To further investigate the relevance of LIF in angiogenesis, we examined the effect of the recombinant mouse Lif on the expression of angiogenic genes in the mouse ECs and PCs. Lif significantly suppressed the expression of angiogenic genes, such as, *Ang2*, *Esm1*, *Dll4, Vegfr2, Sox7, Sox17, and Sox18 in* MS1 (Fig. 3g). In a previous study, these genes were upregulated by members of the Sox family [17], which play important roles in the vascular system development [18]. We found that Lif significantly suppressed Sox17, but not Sox7 or Sox18, in ECs (Fig. 3g). In PCs, Lif suppressed *Vegfa*, *Fgf2*, *Pdgfbb, Dll4, Vegfr2 and Ang2* expression (Fig. 3h). Interestingly, inhibition of palmitoylation by 2-bromopalmitate (2-bp) significantly inhibited the expression of Lif which was induced by Kai1 (Fig. 3i).

**Fig. 4:**
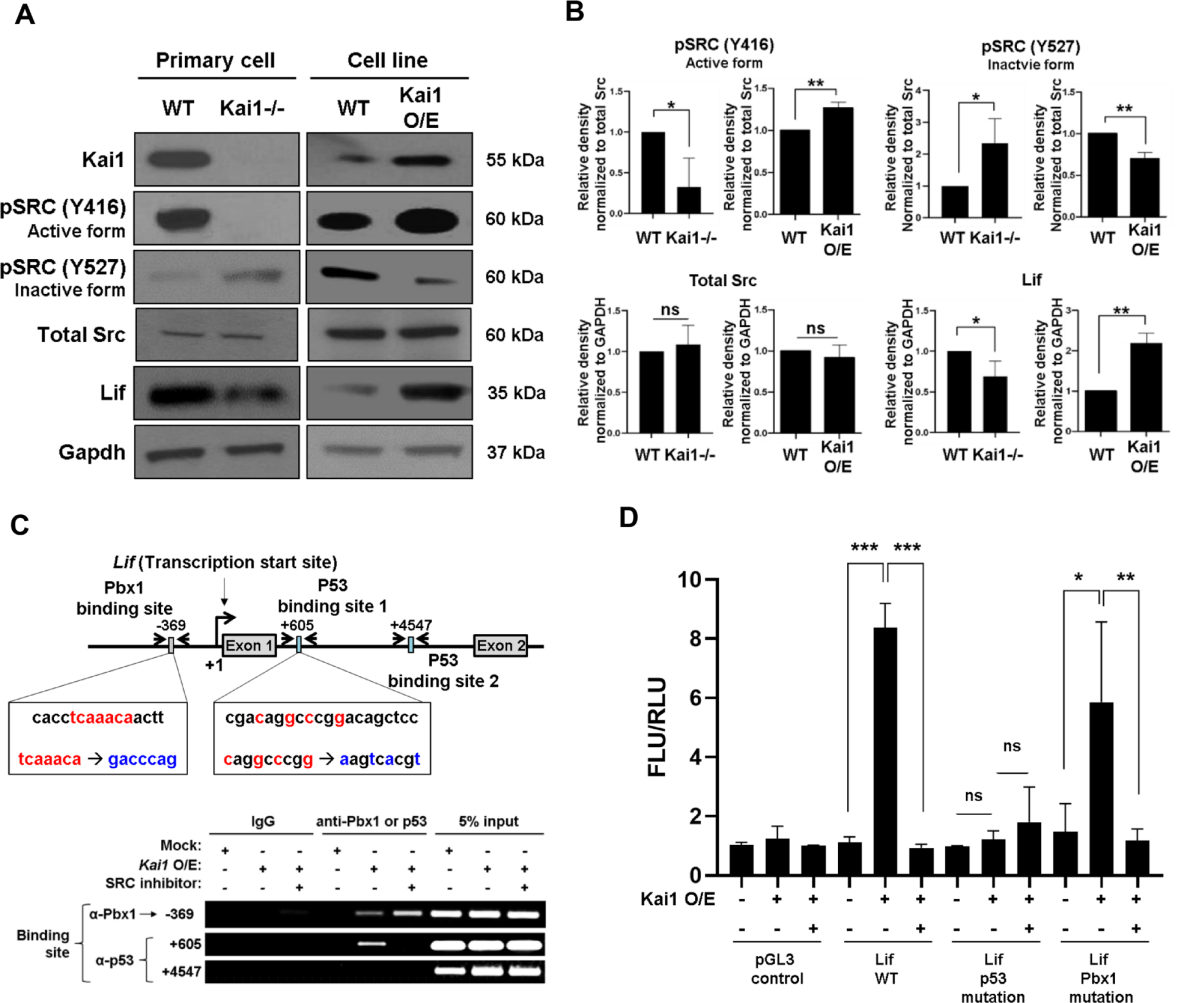
LIF is induced by KAI1 through Src/p53 axis in PCs. **a** Src phosphorylation levels and positions and Lif immunoblotting results in left, WT primary PC and Kai1−/− primary PC and, right, WT 10T1/2 and Kai1-O/E 10T1/2. **b** Quantification of the obtained results (*ns* not significant, *p < 0.01, **p < 0.05, Means ± SEM, 3 technical replicates). **c** Top, scheme, showing the regions targeted with the chromatin immune-precipitation (ChIP) assay and mutagenesis. Bottom, binding of p53 and Pbx1 on *Lif* gene-regulating element or promoter region using ChIP assay. **d** Activation of mouse Lif gene-regulating element or promoter in Kai1-O/E 10T1/2 cells using luciferase assay. *ns* not significant, *p < 0.01, **p < 0.05, ***p < 0.001

Next, we investigated the signalling pathways of the KAI1-LIF axis in PCs. Among the inhibitors of several signalling pathways examined, Src inhibitor nearly completely blocked the induction of *Lif* mRNA by Kai1 O/E in PCs (Additional file 1: Fig. EV4A). In Kai1^−/−^ primary PCs, the active form of Src was decreased (phosphorylation at tyrosine 416 of Src), whereas inactive form of Src was increased (phosphorylation at tyrosine 527 of Src) compared to in WT PCs. In Kai1 O/E PCs, the opposite results were obtained (Fig. 4a, b). To reveal the relevance of the Src at the downstream of KAI1, PP2, the specific inhibitor of Src was used [19, 20]. The supernatant of Kai1-O/E PCs (10T1/2) inhibited endothelial cell tube formation and ECs migration, which was reversed by the Src inhibitor (PP2) (Additional file 1: Fig. EV4 B and C).

And we evaluated the downstream of the KAI1/Src pathway by examining transcription factor-binding sites in the *Lif* promoter region. A consensus p53-binding element in mouse *Lif* gene was previously reported at position + 4547 (p53 binding site 2) [21]. We identified an additional p53-binding element in this gene at position + 605 (p53 binding site 1) from the transcription start site using a prediction program. Additionally, a consensus Pbx1 binding element in mouse *Lif* gene was confirmed using prediction program. Interestingly, we found that Kai1 induced binding of p53 at + 605 position of the *Lif* intron, which was reversed by the Src inhibitor (PP2) (Fig. 4c). In contrast, Pbx1 binding at the *Lif* promoter by *Kai1* O/E was increased by the Src inhibitor (Fig. 4c). To investigate whether the binding of Pbx1 and p53 in the *Lif* gene-regulating element is important for KAI1/Src mediated Lif expression, we performed luciferase assay. Since Kai1 induced the protein level of p53 and Pbx1, it might effect on the enhancement of Lif gene-regulating element’s activation (Additional file 1: Fig. EV4D and E). We also assessed the specific effects of p53 and pbx1 on the *Lif* promoter or gene-regulating element, by constructing a luciferase vector with a mutation in the p53 or pbx1 binding sites, respectively. Interestingly, we found that KAI1 induced luciferase activity in the *Lif* promoter of wild type, which was reversed by the Src inhibitor. And the mutant p53 binding site in the *Lif* gene-regulating element was not activated by Kai1. However, the mutation of Pbx1 binding site did not affect activation of the *Lif* promoter (Fig. 4d). From these results, p53 is the specific transcription factor to activate *Lif* gene-regulating element.

### KAI1 directly binds and sequesters VEGF and PDGF, to inhibit the signalling they trigger via their respective cell surface receptor

**Fig. 5:**
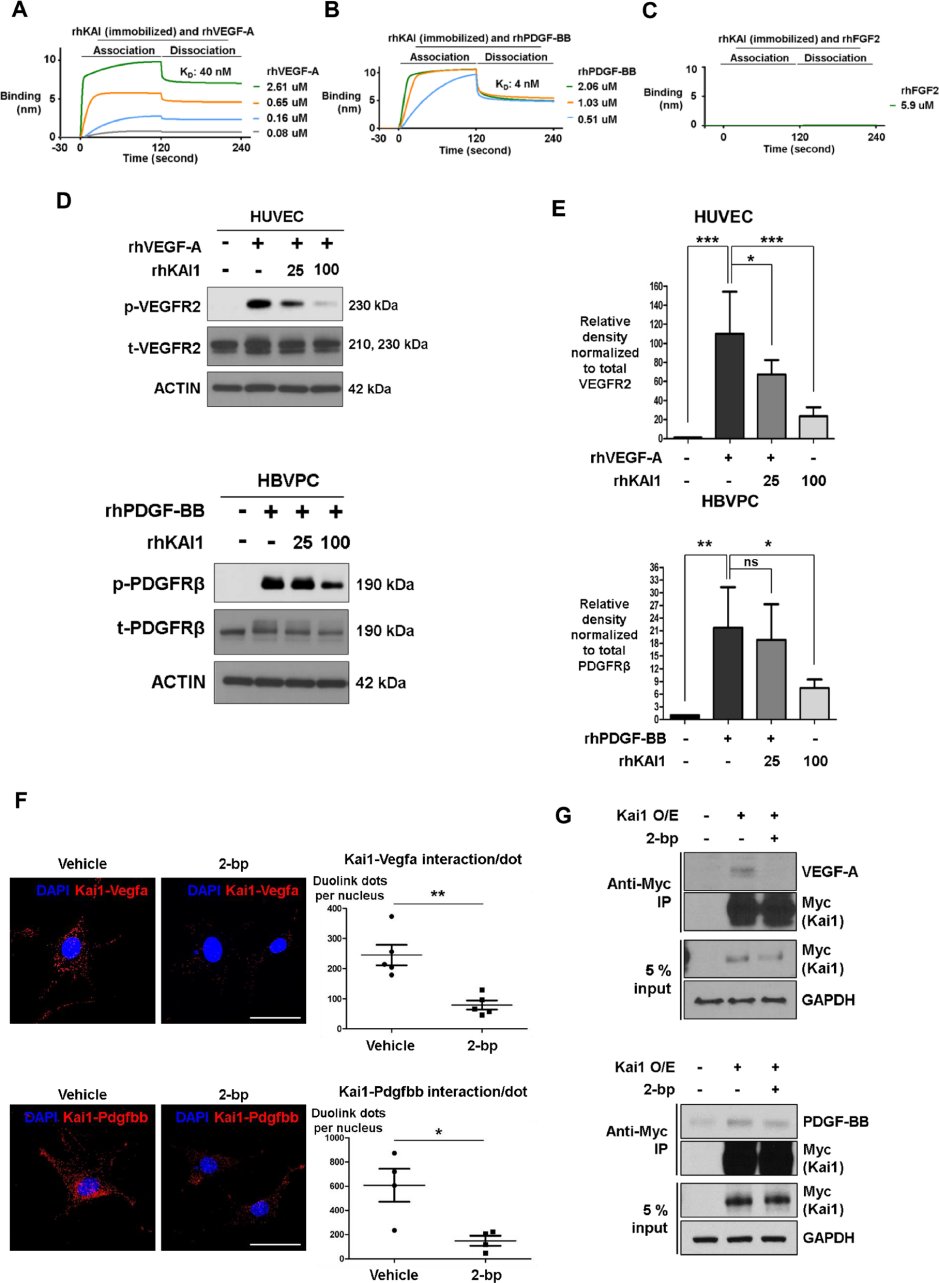
KAI1 binds VEGF-A and PDGF-BB directly, resulting in inhibition of VEGFR2 and PDGFRβ signaling. **a**–**c** Protein–protein interactions between rhKAI1, rhVEGF-A, rhPDGF-BB and rhFGF2 observed using surface plasmon resonance. A shift in nm indicates the binding of two proteins. **d** Top, VEGFR2 phosphorylation levels in HUVECs, in response to rhVEGF-A and rhKAI1 treatment. Bottom, PDGFRβ phosphorylation level in human brain vascular PCs (HBVPs), in response to rhPDGF-BB and rhKAI1 treatment. **e** Quantification of the obtained results (*ns* not significant, *p < 0.01, **p < 0.05, ***p < 0.001, Means ± SEM, 3 technical replicates). **f** Left, Bimolecular fluorescent complementation assays using Duolink visualizes the interaction of Kai1-Vegfa and Kai1-Pdgfbb in living cells. Right, quantification of the obtained results (*p < 0.01, **p < 0.05, Means ± SEM, 3 technical replicates). Scale bar, 50 μm. **g** After palmitoylation inhibition in 293 T cells, protein–protein interactions between KAI1-rhVEGF-A (top) and KAI1-rhPDGF-BB (bottom) observed using the immunoprecipitation (IP) assay

Previously, anti-KAI1 antibody was shown to inhibit VEGF signalling by regulating the KAI1 distribution in the lipid rafts [22]. We predicted that KAI1 binds VEGF or VEGFR directly to inhibit VEGF signalling. We found that recombinant human KAI1 (rhKAI1) bound to recombinant human VEGF (rhVEGF) and rhPDGF-BB, but not rhFGF2 (Fig. 5a–c). KAI1 did not bind to cytokine receptors such as VEGFR2 (Additional file 1: Fig. EV5A). Binding of KAI1 to VEGF or PDGF resulted in sequestration of angiogenic cytokines, leading to inhibition of VEGFR2 and PDGFRβ phosphorylation in HUVECs and HBVPs even in the presence of rhVEGF-A or rhPDGF-BB (Fig. 5d, e). Next, to visualize the interaction of KAI1 and VEGF-A or PDGF-BB in living cells, we performed a bimolecular fluorescent complementation assay based on the complementation between two non-fluorescent fragments of fluorescent protein that are brought together by an interaction between proteins fused to each fragment. These data clearly visualized the direct binding between KAI1 and VEGF-A or PDGF-BB in living cells (Fig. 5f). In addition, we carried out an in vitro co-immunoprecipitation assay with KAI1-Myc and VEGF-A or PDGF-BB. And we confirmed that KAI1 interacted VEGF-A and PDGF-BB. When we treated 2-bp, there was no binding between KAI1 and VEGF-A or PDGF-BB (Fig. 5g). These findings provide the evidence of the requirement of membrane-localized KAI1 for interaction between KAI1 and VEGF-A or PDGF-BB. Additionally, rhKAI1 inhibited endothelial cell tube formation and ECs migration (Additional file 1: Fig. EV5B and C).

Taken together, KAI1 exerts anti-angiogenic effects by two mechanisms. First, KAI1 induces LIF through the Src/p53 axis, which downregulates angiogenic genes. Second, KAI1 directly binds to VEGF-A and PDGF-BB, leading to inhibition of VEGF/PDGF signalling.

### KAI1 has therapeutic potential for inhibiting tumor angiogenesis and growth

To assess the relevance of KAI1 and angiogenesis in tumor, we examined the expression level of KAI1 in human melanoma, prostate cancer and pancreatic cancer tissues by immunohistochemistry. We found that KAI1 was highly expressed in the normal tissues, in contrast, KAI1 level was significantly decreased in cancer tissues (Additional file 1: Fig. EV6A, D, and G). Similar results were obtained by immunofluorescent staining for KAI1 in these tissues (Additional file 1: Fig. EV6B, C, E and F). Interestingly, KAI1 was predominantly expressed in PCs (PDGFRβ^+^) rather than in ECs (VE-cadherin^+^) of the normal tissues (Additional file 1: Fig. EV6B and E).

**Fig. 6:**
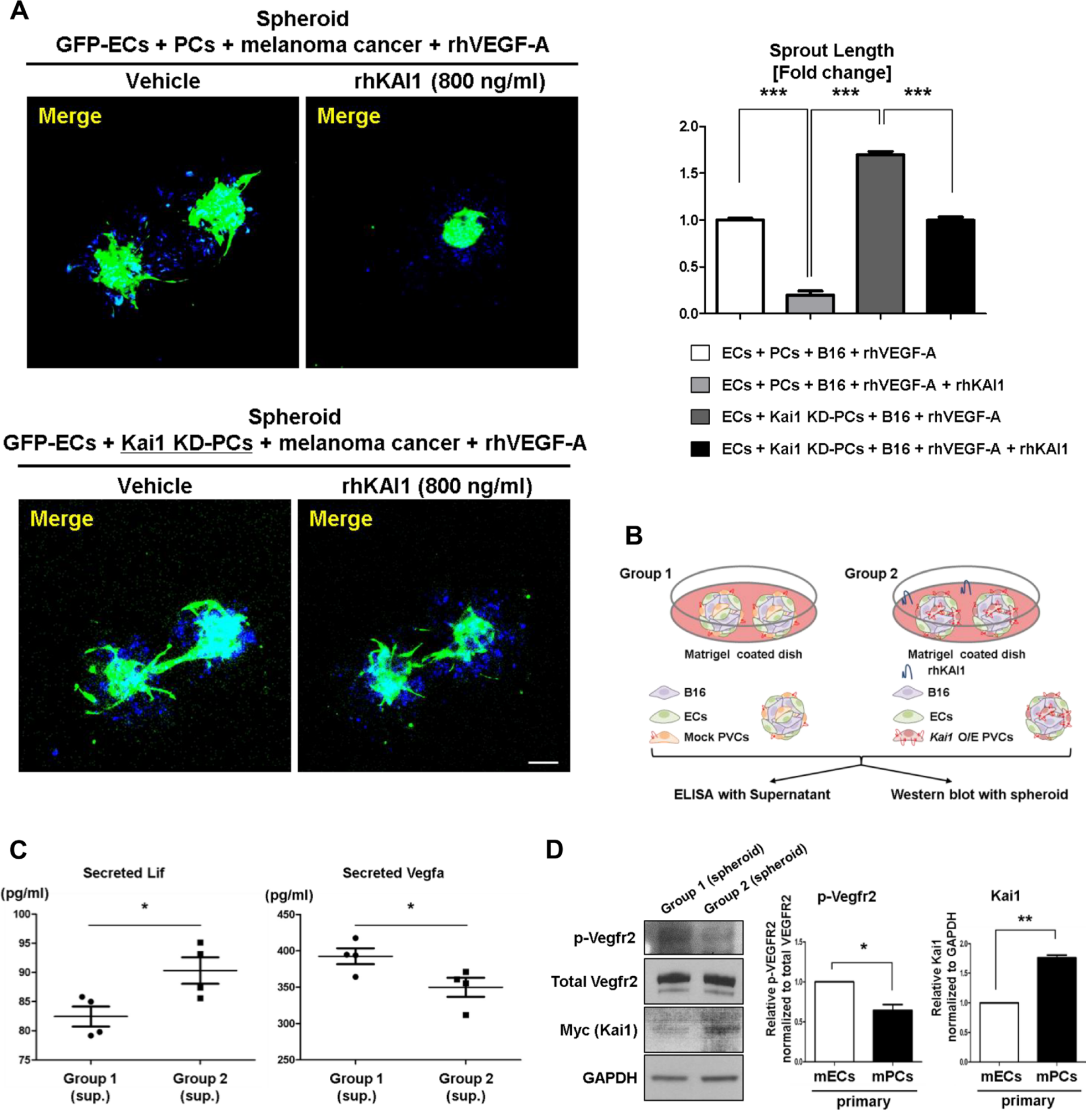
Therapeutic potential of KAI1 to inhibit tumor angiogenesis and growth. **a** Left, In vitro matrigel tube formation assay using cellular spheroid consisting of MS1, 10T1/2 (WT and Kai1-KD), and B16 (melanoma cancer cell line) cells treated with 40 ng/mL of rhVEGF-A following the treatment with rhKAI1 (800 ng/mL). Scale bar, 100 μm. Right, Quantification of the obtained results (*p < 0.01, **p < 0.05, ***p < 0.001, Means ± SEM, 3 technical replicates). **b** Overview of experiment design applied to ELISA and western blot. **c** ELISA assay measuring the levels of mouse Lif and mouse Vegfa secreted into the media of three-hybrid spheres consisting of cancer (melanoma cells) and blood vessel cells, namely primary EC, as well as primary PC transfected with a mock vector (Group 1) and PC overexpressing Kai1 (Group 2). **d** Left, Vegfr2 phosphorylation levels and Kai1 immunoblotting results in group 1 and 2. Right, quantification of the obtained results (*p < 0.01, **p < 0.05, Means ± SEM, 3 technical replicates)

To investigate the therapeutic potential of KAI1 against tumor growth, we first examined the anti-angiogenic role of rhKAI1 using three-dimensional hybrid spheres consisting of cancer and blood vessel cells (melanoma cells; B16 (mouse melanoma cancer cells), ECs; MS1, and PCs; 10T1/2). Spheres treated with rhKAI1 showed a reduced sprouting activity compared to cells treated with vehicle (Fig. 6a). Interestingly, spheres including KAI1 knockdown PC showed the most angiogenic phenotype and spheres including wild-type PC treated with rhKAI1 showed the most anti-angiogenic.

**Fig. 7:**
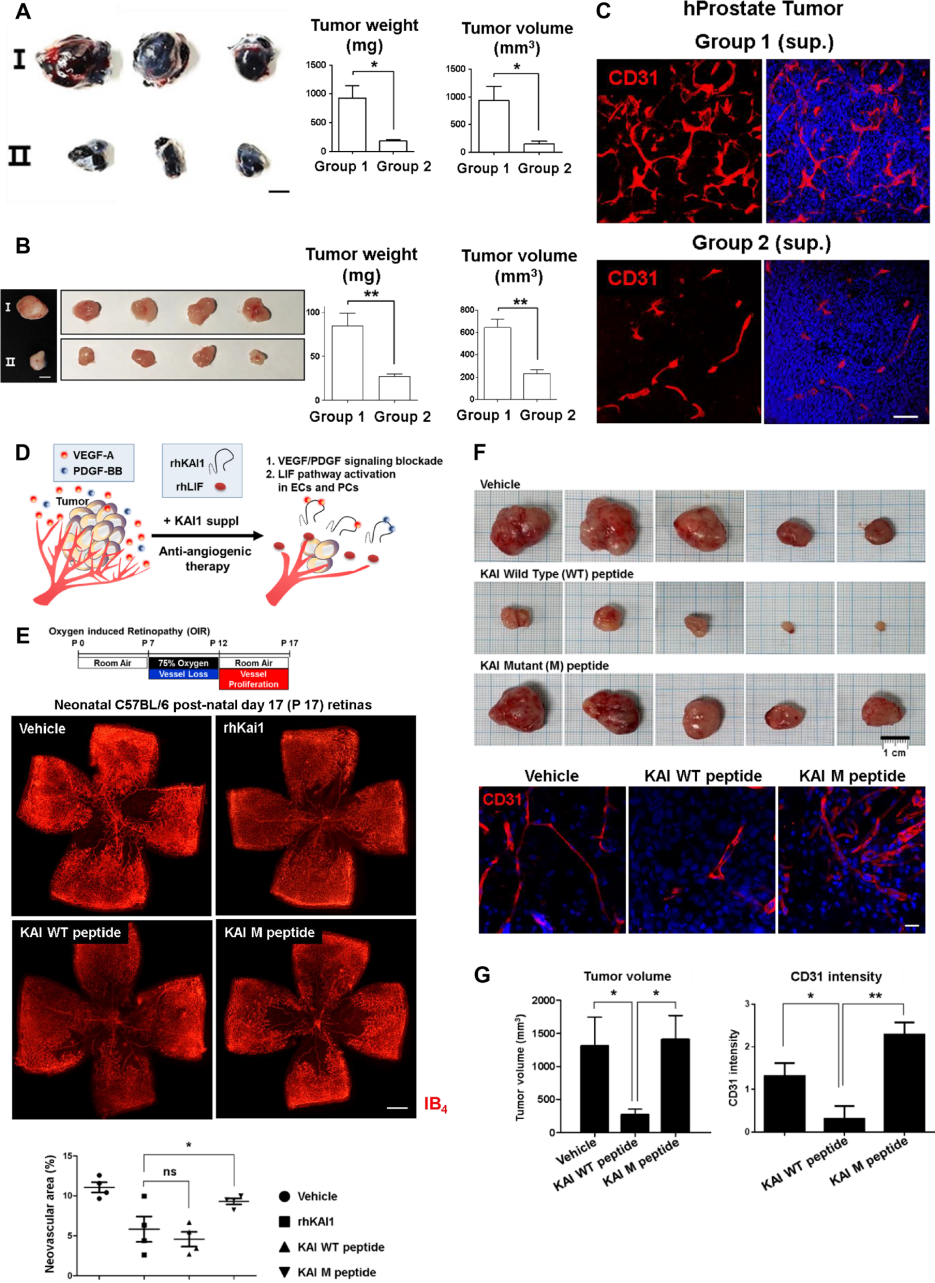
Therapeutic potential of KAI1 and peptide to inhibit angiogenesis in tumors and prevent OIR-induced neovascularization. **a** B16 cells were subcutaneously injected in C57BL/6 mice with or without KAI1 supplement [combination of [1] Kai1-O/E 10T1/2 supernatant to provide anti-angiogenic LIF and [2] rhKAI1 to directly bind and inhibit VEGF and PDGF]. Left, tumor growth images are presented. Scale bar, 5 mm. Right, quantification of the obtained results (Means ± SEM, 3 biological replicates). **b** PC3 (human prostate cancer cell line) cells were subcutaneously injected into NSG mice in combination with or without KAI1 supplement. Left, tumor growth images are presented. Scale bar, 5 mm. Right, quantification of the obtained results (Means ± SEM, 5 biological replicates). **c** CD31 (red) immunostaining, and DAPI (blue) staining of human prostate (PC3) tumors treated with or without KAI1 supplement. Scale bar, 100 μm. **d** Schematic figure of dual anti-angiogenic effect of KAI1 in tumors. **e** Neonatal C57BL/6 mice with OIR at postnatal day 15 (P 15) were treated or not treated with a single intravitreal injection (1 μL) of vehicle only [PBS] (*n* = 4 retinas), rhKAI1 (600 ng), wild-type peptide KAI WT (200 ng) (*n* = 4 retinas), or mutant peptide KAI M (200 ng) (*n* = 4 retinas). Whole-mount retinas were stained with isolectin-B4 conjugated to Alexa Fluor 594 red-fluorescent dye. Top, Representative confocal microscopy images of retinas of OIR neonatal C57BL/6 mice at P17. Bottom, quantification of neovascularization in the retinas of OIR neonatal C57BL/6 mice treated or not treated with rhKAI1, KAI WT or KAI M peptides. Scale bar, 500 μm. **f** Mice were injected with matrigel and MDA-MB 231 cells in the presence of PBS alone (vehicle) or 200 ng KAI WT or M peptide (*n* = 5). After 23 days, the plugs were removed and the angiogenic responses were evaluated. Top, tumor volume was presented and bottom, representative IF images of tumors are shown. **g** Quantitative results of tumor volume (left) and CD31 intensity (right). Endothelial cells were visualized with CD31 (red) immunostaining, and DAPI (blue) staining of human breast cancer (MDA-MB231) treated with KAI WT or KAI M peptides. Scale bar, 20 μm. *ns* not significant, *p < 0.01, **p < 0.05, ***p < 0.001

To investigate the therapeutic potential of KAI1 for inhibiting cancer growth in an in vivo mouse model, we subcutaneously transplanted B16 and PC3 (human prostate cancer cells) with and without KAI1 supplementation (combination of two strategies, firstly, rhKAI1 to bind and sequester VEGF or PDGF and, secondly, supernatant from *Kai1*-O/E PCs containing Lif to inhibit angiogenesis). Before transplant cancer cells into the mouse, we determined the Lif mediates Kai1 function in this system. First, we measured the secreted Lif level in the media of three-dimensional hybrid spheres. We detected more secreted Lif in Kai1 overexpressed group. In contrast, the Vegfa level was decreased in Kai1 overexpressed group (Fig. 6b, c). Interestingly, the phosphorylation of Vegfr2 was also decreased in Kai1 overexpressed group (Fig. 6d). These findings provide the evidence of LIF mediated KAI1 function in anti-angiogenesis. In vivo, melanoma and prostate tumor growth was remarkably retarded by supplementation with KAI1 (Fig. 7a, b). According to histologic examination, vessel formation in tumors was significantly decreased by supplementation with KAI1 (Fig. 7c). However, KAI1 did not effect on the proliferation of melanoma cells (Additional file 1: Fig. EV7). This means that KAI1 reduced the tumor growth through inhibition of angiogenesis, not through inhibition of proliferation (Fig. 7d). Interestingly, when we transduced the expression of *Kai1* gene in mouse Tibialis Anterior muscle, we could detect the Kai1 and Lif simultaneously in the same location (Additional file 1: Fig. EV8A and B). Therefore, KAI1 shows potential for preventing cancer angiogenesis and tumor growth by gene therapy.

Large extracellular loop (LEL) of KAI1 has been demonstrated to be essential for at least some of the biological activities of tetraspanins [23]. In this study, we demonstrated that KAI1 interact with VEGF and PDGF but not with FGF. To design peptide which interact with VEGF and PDGF, we screened the consensus amino acid sequences of VEGF- and PDGF-receptor but exclusive with FGF-receptor sequence. Since domain 2, 3 and 4 of these two receptors are known as essential for ligand binding [24], we narrowed down to these sequences. Lastly, we confirmed these sequences are matched in amino acids sequences of LEL of KAI1 (Additional file 1: Fig. EV9A and B). And we found that 166–185 amino acids of KAI1 protein matched this condition and confirmed that this peptide interact with VEGF and PDGF as much as recombinant KAI1 (Additional file 1: Fig. EV10). We also constructed mutant peptide which are substituted predicted binding sites with alanine and the mutant peptide did not interact with VEGF and PDGF (Additional file 1: Fig. EV10). Surprisingly, a 20-mer peptide derived from the large extracellular loop (LEL) of KAI1 has been shown to have anti-angiogenic effects to block retinal neovascularization (Fig. 7e) and the progression of breast cancer in vivo (Fig. 7f, g). However, mutant peptide did not show any effect in this in vivo models. According to histologic examination, vessel formation in tumor was significantly decreased by KAI peptide (Fig. 7f, g).

## Discussion

We demonstrated that the KAI1/CD82 is a key molecule to controlling angiogenesis and switching angiogenic milieu to a quiescent state. When a quiescent situation is required and induced, KAI1 is palmitoylated by zDHHC4 and localizes to the lipid raft membrane of PCs, induces transcription of *Lif* through the Src/p53 axis, and leads to downregulation of angiogenic genes in PCs (*Vegfa, Fgf2*, *Pdgfbb, Dll4, Vegfr2 and Ang2*) and in ECs (*Sox17, Ang2*, *Esm1*, *Dll4,* and *Vegfr2*). Finally, KAI1 inhibits angiogenesis. Another anti-angiogenic mechanism of KAI1 is its binding to VEGF-A and PDGF-BB, which leads to inhibition of phosphorylation or activation of VEGFR2 or PDGFRβ. Finally, we demonstrated the therapeutic potential of KAI1 using an in vivo diseases model by showing that angiogenesis can be suppressed by treatment with recombinant or peptide of KAI1.

Previous studies of the function of KAI1 mostly focused on activation of T cells and inhibition of metastasis and migration through suppression of Rac1/RhoA activity in the cancer cells [25, 26]. Recently, we and other groups reported that KAI1 regulates the cell cycle of long-term repopulating hematopoietic stem cells (LT-HSCs) and muscle stem cells, respectively [5, 6]. Here, we demonstrated another important function of KAI1; we found that PCs control angiogenesis and that KAI1 in PCs is a key molecule involved in inhibiting angiogenesis.

During our investigation of how KAI1 to regulates angiogenesis, a paper [27] was published by Wei et al*.* who demonstrated that a lack of *Kai1* in endothelial cells promotes angiogenesis in a pathological state. However, they did not identify the vascular cells that predominantly express KAI1*.* Based on this research, we investigated the main cells expressing KAI1 and the underlying mechanism of how KAI1 inhibits angiogenesis in the complex network between vascular cells. We generated *Kai1*-*GFP* transgenic mice and found that PCs, but not ECs, are the main cells expressing KAI1 among vascular cells. We demonstrated that ECs express KAI1 very weakly and that *Kai1* overexpression in ECs did not significantly inhibit angiogenesis, whereas KAI1 in PCs efficiently inhibited angiogenesis. We next determined the underlying mechanisms of how KAI1 on PCs control ECs to suppress angiogenesis.

In the *Kai1-GFP* mice model, we confirmed that KAI1 is expressed not only in PCs, but also in leukocytes and erythrocytes. Previous studies also reported KAI1 expression in T cells, B cells, M1-like macrophages, and erythrocytes [5, 25, 28]. However, the role of KAI1 remains unknown in macrophages, erythrocytes, and B cells, except for in T cell activation. The role of KAI1 on leukocytes and erythrocytes requires further analysis.

The most important difference in the intracellular distribution of KAI1 was that KAI1 was observed in the membrane layer of PCs but not in ECs, possibly because of palmitoylation of KAI1. Palmitoylation of KAI1 is observed mainly in the PCs but not in ECs. After screening of 23 enzymes in the zDHHC family, zDHHC4 was found to be the palmitoyltransferase responsible for the palmitoylation of KAI1. The recently discovered zDHHC4 molecule has not been widely studied [29], and its role in vascular diseases should be further evaluated. Palmitoylation of proteins prevents ubiquitination-mediated protein degradation [30, 31]. Thus, palmitoylation of KAI1 by zDHHC and its localization in lipid raft may increase its stability, leading to anti-angiogenic effects.

To identify the distal effector molecule of KAI1 in PCs, we analyzed the transcriptome of WT and *Kai1*^−/−^ PCs, performed GSEA, and found LIF is the downstream effector of KAI1. We demonstrated that both EC and PC express LIF receptors, suggesting that an autocrine and paracrine network of KAI1/LIF and angiogenic factors functions between these cells.

Tetraspanin is commonly considered as a molecular facilitator that induces signalling by assisting other molecules without directly signalling itself, as it does not possess a signalling motif [25]. Recently, however, there is an interesting article that suggested that the tetraspanin molecule, CD37, is directly involved in signal transduction [32]. To evaluate the signalling pathway between KAI1 and LIF in PCs, we tested inhibitors of several major pathways and found that KAI1 induced LIF via Src stimulation in PCs. In several studies, overexpression of Kai1 was shown to cause transduction of multiple signals such as in SRC signalling [25]. It has also been reported that KAI1 molecules bind to each other and activate SRC when treated with KAI1 antibody [33]. Thus, *Kai1* is thought to activate SRC through the same mechanism by binding of KAI1 proteins to each other or by enhancing interactions with other tetraspanin molecules with high expression in PCs.

We found that the transcription factor p53 enhances LIF expression in response to KAI1 stimulation. Interestingly, p53 binds to the gene-regulating element of *Lif* through SRC signalling activated by KAI1, whereas Pbx1 binds to the promoter region of *Lif* independently of SRC signalling. Binding of Pbx1 to the *Lif* promoter may be facilitated via SRC-independent signalling by KAI1 or other signalling molecules of the tetraspanin-enriched web activated by KAI1. Moreover, the pbx1 binding site mutation did not affect activation of the Lif promoter. Here, we demonstrated the potential anti-angiogenic functions of p53 such as increasing LIF in response to KAI1. This new role for p53 in vascular cells and cancer cells requires further analysis.

Tetraspanins are distributed in a broad tissues and enriched in the membrane of exosome [34]. Exosome has been deciphered to participate in cellular communication through the shuttling of bioactive miRNAs, proteins, and mRNAs. While miRNAs do not code for proteins, they can modulate expression of target proteins by regulating the degradation or translation of their targeted mRNA. Emerging evidence shows that the progression and metastasis of human cancers are mediated by dysregulation of miRNAs [35]. Inducing angiogenesis is also regulated by miRNAs [35]. So, exosome is another valuable messenger to control angiogenesis through miRNA. To investigate the role of KAI1 in angiogenesis through the exosome, we compared miRNAs in exosome derived from wild-type or KAI1-knockout aorta. We performed quantitative real-time PCR and found that miR181a (known as inducing angiogenesis [36, 37]) level was higher in exosome derived from KAI1 knockout aorta. In contrast, miR107 (known as suppressing angiogenesis [38]) level was much lower in exosome derived from KAI1 knockout aorta (Additional file 1: Fig. EV11). Based on this result, we are planning on investigating the mechanism how KAI1 regulate the level of miRNAs. And also we are going to screen miRNAs regulated by KAI1.

Downstream of LIF, our study revealed that LIF from PCs or recombinant LIF inhibited the expression of various angiogenic factors in the neighboring cells (*Ang2*, *Esm1*, *Dll4*, *Vefgr2* and *Sox17* in EC and *Vegf*, *Fgf2*, *Pdgfbb, Dll4, Vegfr2, and Ang2* in PC). Notably, key regulators of angiogenesis in ECs were inhibited, such as *Sox17* which enhances VEGF signalling in a positive feedback loop and increases the tip cell-related genes essential for angiogenesis [39]. Thus, inhibition of the *Sox17* expression in ECs by the KAI1-LIF axis in PCs may have potent anti-angiogenic effects in ECs by blocking VEGF signalling through multiple aspects in the angiogenic microenvironment.

Analysis of the relationship between KAI1 and growth factor signalling showed that KAI suppressed VEGF expression via Src activation in prostate cancer cells [40]. Ectopic KAI1 expression in renal cell carcinoma suppressed TGF-β1 signalling, leading to inhibition of migration and invasion [41]. In this study, the very interesting finding is that KAI1 used another mechanism to inhibit growth factors and angiogenesis, which involved direct binding to and inhibition of VEGF and PDGF, but not FGF. VEGF is known to be structurally very similar to PDGF, and recent reports showed that PDGF can use VEGF-R2 as its receptor [42]. Therefore, we expected that there are the common motif of VEGF and PDGF which binds to VEGFR2 and the sequence and structure of the KAI1 molecule involved in binding and inhibition of VEGF and PDGF. In this study, we identified the peptide from the anti-angiogenic sequence of KAI1 which bind and quench VEGF and PDGF simultaneously. It may lead to the development of therapeutic agents for cancer or various vascular diseases. Human VEGF-A has eight exons and seven introns, and alternative exon splicing of VEGF-A results in the production of four different isoforms: VEGF121, VEGF165, VEGF189, and VEGF206. There are three VEGF receptors VEGFR1, VEGFR2, and Neurophilin-1/Neurophilin-2, which have various affinities for VEGF [43]. Increased VEGF expression is mostly related to pathological angiogenesis affecting the proliferation of cancer cells [44]. Although VEGF_165_ used in this study is the predominant isoform, further studies are needed to determine whether these various VEGF proteins bind to KAI1 protein.

In most of the previous studies, the anti-cancer effect of KAI1 was evaluated using *Kai1* over-expressing cancer cells [40, 45]. In this study, we demonstrated the feasibility of using KAI1 as the anti-cancer therapeutic agent by KAI1-supplementation involving two components: (1) recombinant protein or peptide of KAI1 binds to and sequesters VEGF or PDGF, and (2) the supernatant of *Kai1*-O/E PCs containing anti-angiogenic LIF. The anti-cancer effect by KAI1 supplementation show potential for drug development.

We identified a novel endogenous switch, KAI1, which turns angiogenesis off and is mainly expressed in PCs. This protein triggers the quiescent milieu by inducing the pivotal anti-angiogenic molecule LIF or by directly binding to and inhibiting VEGF and PDGF. These findings provide a new platform for mechanistic studies of vessel homeostasis. Our results demonstrate the importance of PCs in controlling ECs in the context of angiogenic equilibrium.

## Conclusions

Understanding angiogenesis is essential to develop a therapeutic modality for cancer or ischemic cardiovascular diseases. However, little is known about endogenous inhibitors of angiogenic growth factors such as VEGF and PDGF. Pericytes are known as helper to stabilize the vessel wall and prevent vascular leakage. However, the functional roles of pericytes in anti-angiogenesis are currently not fully understood. In this study, we investigated the role of Kai1 on the regulation of angiogenesis. By making Kai1 knockout mice, we confirmed that Kai1 knockout facilitated angiogenesis like neonatal retina demonstrating it is anti-angiogenic. After extensive mechanism studies, we found that KAI1 induced LIF (leukemia inhibitory factor) through Src/P53 pathway in pericytes. LIF from pericytes turns down several angiogenic genes in ECs (Sox17 and others) as well as in pericytes themselves under paracrine and autocrine way. Intriguingly, Kai1 had another anti-angiogenic mechanism that it directly bound to VEGF or PDGF and inhibited their signalling. Clinical applicability was well confirmed by two different cancer-angiogenesis in vivo models, where Kai1 supplement significantly suppressed tumor angiogenesis and growth.

Finally, a 20-mer peptide derived from the large extracellular loop (LEL) of KAI1 has been shown to have anti-angiogenic effects to block retinal neovascularization and the progression of breast cancer in vivo.

1. We found that KAI1 in pericytes as the new endogenous counter-regulator of pro-angiogenic factors such as VEGF and PDGF.
2. Detailed mechanism how Kai1 in pericytes inhibits angiogenesis, would be the important new knowledge that can be basis for the future drug development to control angiogenesis.
3. The finding that KAI1/LIF from pericytes inhibits the expression of angiogenic genes in ECs leading inhibition of angiogenesis, clearly demonstrates the paracrine mechanism of angiogenesis and the importance of pericytes.
4. Evidence of direct binding between KAI1 to VEGF and PDGF may open the new research field of biology around angiogenic growth factors as well as development of multi-target inhibitor against angiogenic growth factors.
5. The findings from cancer-angiogenesis in vivo models suggest that the peptide derived from KAI1 may provide the promising future platform of new drug development for cancer or other vascular proliferative disease.

## Supplementary Information

**Additional file 1: Figure EV1.** KAI1 was expressed in PC more than EC and plays a role in the anti-angiogenic effect in PC. (A) Left, vessel regression (empty sleeves, BS-1 lectin-negative, collagen IV-positive) in the WT and *Kai1−/−* mice. Arrowheads, empty sleeves. Scale bar, 50 μm. Right, quantification of the obtained results (Means ± SEM, 4 technical replicates). (B) Confocal imaging in retinal of WT mice using Kai1 (white) antibody. Scale bar, 20 μm. (C) Confocal imaging of Kai1-GFP expression in brain and heart of the Kai1-GFP fusion transgenic mice. Scale bar, 5 μm. (D) FACS plot showing primary ECs (CD45^−^/CD31^+^) and primary PCs (CD45^−^/Pdgfrβ^+^) in the bone marrow of Kai1-GFP fusion transgenic mice. Left, KAI1 surface expression of human cell lines, HUVECs and HBVP, analyzed with FACS. Right, quantification of the obtained results. MFI (**p < 0.05, mean fluorescence intensity). (F) CD9 expression as an example in MS1 and 10T1/2 cells. Scale bar, 50 μm. (G) Expression of tetraspanins (Cd9, Cd81, Cd151) in ECs (MS1) and PCs (10T1/2) by using quantitative RT-PCR. (H) In vitro matrigel tube formation assay, performed using co-culture spheroids, consisting of ECs (MS1) and PCs (10T1/2), transduced with the indicated adenovirus at 0, 14, and 107 h, and observed using time-lapse microscopy. Scale bar, 50 μm. **Figure EV2.** KAI1 localizes at the lipid rafts of PCs and binds with zDHHC3 and zDHHC4. (A) Lipid rafts isolated from ECs (MS1) and PCs (10T1/2) using OptiPrep medium (frozen and crosslinks method). Endogenous Kai1 localization is confirmed. Flotillin-1 (Flot-1) and caveolin-1 (Cav-1) was detected lipid raft membrane fraction of MS1 and 10T1/2, respectively. Fraction 1; no cellular protein, Fraction 2; Lipid raft proteins. Fraction 3; Non-raft membrane proteins. Fraction 4 and 5; Cytosolic proteins. (B) Semi-quantitative RT-PCR analysis for *zDHHC* family member expression in MS1 and 10T1/2 cells. The genes were normalized by *Gapdh* (Means ± SEM, 3 technical replicates). (C) zDHHC3 and zDHHC4 binding to KAI1, confirmed by co-immunoprecipitation. **Figure EV3.** Identification of the downstream effect molecule induced by KAI1. (A) Semi-quantitative RT-PCR analysis for mRNA levels of top 17 genes in “negative angiogenic regulators” in WT and Kai1−/− primary PCs. The genes were normalized by *Gapdh* and the mRNA expression value are as fold change relative to WT (Means ± SEM, 3 or 4 technical replicates). (B) Mouse EC cell line, MS1, cell cycle analyzed with FACS (ns; not significant, *p < 0.01, **p < 0.05, Means ± SEM, 3 technical replicates). (C) *LIF*, *LIFR*, and *GP130* expression in mouse and human ECs and PCs by using semi-quantitative RT-PCR. **Figure EV4.** KAI1 induces LIF through Src/p53. (A) *Lif* and *Kai1* mRNA expression in 10T1/2 cells pre-treated with inhibitor of p38, Erk, Pkc, or Src and then transfected with Kai1-O/E using Flag-Kai1 vector. (B) The effects of Kai1 overexpression sup. and PP2 (Src inhibitor) on angiogenesis using tube formation assay (***p < 0.001, Means ± SEM, 3 technical replicates). (C) The effects of Kai1 overexpression sup. and PP2 (Src inhibitor) on angiogenesis using migration assay (ns; not significant, ***p < 0.001, Means ± SEM, 3 technical replicates). (D) Protein level of p53 and Pbx1 by Kai1 O/E in 10T1/2 cells. The quantification of the obtained results (*p < 0.01, Means ± SEM, 3 technical replicates). **Figure EV5.** KAI1 inhibits VEGFR2 and PDGFRβ signaling through direct binding to VEGF-A and PDGF-BB. (A) Protein-protein interactions between rhKAI1 and rhVEGF-R2 observed using surface plasmon resonance (BLItz system), which suggests no binding between KAI1 and VEGF-R2. (B) The effects of KAI1 on angiogenesis using tube formation. (C) The effects of KAI1 on angiogenesis using migration. rhVEGF-A (40 ng/ml), rhKAI1 (800 ng/ml) were used. ns; not significant, *p < 0.01, **p < 0.05, ***p < 0.001. **Figure EV6.** The expression of KAI1 proteins in human prostate cancer and melanomas. (A, D) Representative immunohistochemistry staining for KAI1/CD82 protein expression in normal/malignant prostate cancer tissue and normal skin/melanomas. Scale bar, 50 μm. (B, E) Representative fluorescence images of the immunostained normal prostate and skin. (C, F) Representative fluorescence images of the immunostained malignant prostate cancer tissue and melanomas. Scale bar, 8 μm. DIC (Differential interference contrast). (G) Representative immunohistochemistry staining for KAI1/CD82 protein expression in normal/tumor pancreatic tissue. Scale bar, 100 μm. **Figure EV7.** Identification of the role of B16 proliferation by Kai1. WST1 assay measuring B16 cell proliferation rate after Kai1 overexpression .**Figure EV8.** The anti-angiogenic effect through Lif induction in vivo by Kai1. (A) Immunofluorescence image after transducing the Kai1-Flag vector into the mouse TA muscle and detected co-localized Kai1 and Lif. (B) Quantification of the obtained results (Means ± SEM, 4 technical replicates). **p < 0.05, Scale bar, 100 μm. **Figure EV9.** Therapeutic potential of KAI1 and characteristics of KAI1 peptide. (A) Venn diagram showing the design of peptide which interact with VEGF and PDGF, we screened the consensus amino acid sequences of VEGF- and PDGF-receptor but exclusive with FGF-receptor sequence in KAI1 large extracellular loop. (B) Schematic figure showing the KAI 1 peptide and characteristics. **Figure EV10.** Wild-type peptide of KAI1 interact with VEGF and PDGF. Mutant peptide does not interact with them. (A) Peptide-protein interactions between KAI1 peptide and VEGF observed using surface plasmon resonance (BLItz system), which suggests binding between KAI1 WT peptide and VEGF but no binding between mutant peptide and VEGF. (B) Peptide-protein interactions between KAI1 peptide and PDGF observed using BLItz system, which suggests binding between KAI1 WT peptide and PDGF but no binding between mutant peptide and PDGF. **Figure EV11.** Comparative analysis of exosome miRNAs in the aorta of WT and Kai1 knockout mice. Left, Comparison of expression pattern of miR-181a in exosomes of WT and Kai1 knockout mice. Right, Comparison of the expression pattern of miR-107 in exosomes of WT and Kai1 knockout mice. *p < 0.01. Expanded View movie 1. KAI1 in PCs has anti-angiogenic effects. In vitro matrigel tube formation assay, performed using co-culture spheroids, consisting of ECs (MS1) and PCs (10T1/2), transduced with the indicated adenovirus at 0, 14, and 107 h, and observed using time-lapse microscopy. (A) Mock virus transduced MS1 and 10T1/2 co-culture spheroids. (B) Kai1-O/E MS1 and 10T1/2 co-culture spheroids. Video sprouting from the spheroid. Scale bar, 50 μm.

## Data Availability

All data used and/or performed in this study are available from the corresponding author upon reasonability.

## Abbreviations

VEGF: Vascular endothelial growth factor
PDGF: Platelet-derived growth factor
FGF: Fibroblast growth factor
zDHHC: Zinc finger DHHC
LIF: Leukemia inhibitory factor
EC: Endothelial cell
PC: Pericyte
HUVEC: Human umbilical vein endothelial cell

## References

[1] Lechertier T, Reynolds LE, Kim H, Pedrosa AR, Gomez-Escudero J, Munoz-Felix JM, Batista S, Dukinfield M, Demircioglu F, Wong PP (2020). Pericyte FAK negatively regulates Gas6/Axl signalling to suppress tumour angiogenesis and tumour growth. Nat Commun.

[2] Potente M, Gerhardt H, Carmeliet P (2011). Basic and therapeutic aspects of angiogenesis. Cell.

[3] Kim B, Boo K, Lee JS, Kim KI, Kim WH, Cho HJ, Park YB, Kim HS, Baek SH (2010). Identification of the KAI1 metastasis suppressor gene as a hypoxia target gene. Biochem Biophys Res Commun.

[4] Liu WM, Zhang XA (2006). KAI1/CD82, a tumor metastasis suppressor. Cancer Lett.

[5] Hur J, Choi JI, Lee H, Nham P, Kim TW, Chae CW, Yun JY, Kang JA, Kang J, Lee SE (2016). CD82/KAI1 maintains the dormancy of long-term hematopoietic stem cells through interaction with Darc-expressing macrophages. Cell Stem Cell.

[6] Alexander MS, Rozkalne A, Colletta A, Spinazzola JM, Johnson S, Rahimov F, Meng H, Lawlor MW, Estrella E, Kunkel LM (2016). CD82 Is a marker for prospective isolation of human muscle satellite cells and is linked to muscular dystrophies. Cell Stem Cell.

[7] Saeed A, Park R, Sun W (2021). The integration of immune checkpoint inhibitors with VEGF targeted agents in advanced gastric and gastroesophageal adenocarcinoma: a review on the rationale and results of early phase trials. J Hematol Oncol.

[8] Negri S, Faris P, Rosti V, Antognazza MR, Lodola F, Moccia F (2020). Endothelial TRPV1 as an emerging molecular target to promote therapeutic angiogenesis. Cells.

[9] Bergers G, Benjamin LE (2003). Tumorigenesis and the angiogenic switch. Nat Rev Cancer.

[10] Nyberg P, Xie L, Kalluri R (2005). Endogenous inhibitors of angiogenesis. Cancer Res.

[11] Yadav L, Puri N, Rastogi V, Satpute P, Sharma V (2015). Tumour angiogenesis and angiogenic inhibitors: a review. J Clin Diagn Res.

[12] Zhou B, Liu L, Reddivari M, Zhang XA (2004). The palmitoylation of metastasis suppressor KAI1/CD82 is important for its motility- and invasiveness-inhibitory activity. Cancer Res.

[13] Termini CM, Lidke KA, Gillette JM (2016). Tetraspanin CD82 regulates the spatiotemporal dynamics of PKCalpha in acute myeloid leukemia. Sci Rep.

[14] Allen JA, Halverson-Tamboli RA, Rasenick MM (2007). Lipid raft microdomains and neurotransmitter signalling. Nat Rev Neurosci.

[15] Subramanian A, Tamayo P, Mootha VK, Mukherjee S, Ebert BL, Gillette MA, Paulovich A, Pomeroy SL, Golub TR, Lander ES (2005). Gene set enrichment analysis: a knowledge-based approach for interpreting genome-wide expression profiles. Proc Natl Acad Sci USA.

[16] Kubota Y, Hirashima M, Kishi K, Stewart CL, Suda T (2008). Leukemia inhibitory factor regulates microvessel density by modulating oxygen-dependent VEGF expression in mice. J Clin Investig.

[17] Lee SH, Lee S, Yang H, Song S, Kim K, Saunders TL, Yoon JK, Koh GY, Kim I (2014). Notch pathway targets proangiogenic regulator Sox17 to restrict angiogenesis. Circ Res.

[18] Goveia J, Zecchin A, Rodriguez FM, Moens S, Stapor P, Carmeliet P (2014). Endothelial cell differentiation by SOX17: Promoting the tip cell or stalking its neighbor instead?. Circ Res.

[19] Peng Z, Raufman JP, Xie G (2012). Src-mediated cross-talk between farnesoid X and epidermal growth factor receptors inhibits human intestinal cell proliferation and tumorigenesis. PLoS ONE.

[20] Yang C, Zhang M, Sung J, Wang L, Jung Y, Merlin D (2020). Autologous exosome transfer: a new personalised treatment concept to prevent colitis in a murine model. J Crohns Colitis.

[21] Hu WW, Feng ZH, Teresky AK, Levine AJ (2007). p53 regulates maternal reproduction through LIF. Nature.

[22] Nomura S, Iwata S, Hatano R, Komiya E, Dang NH, Iwao N, Ohnuma K, Morimoto C (2016). Inhibition of VEGF-dependent angiogenesis by the anti-CD82 monoclonal antibody 4F9 through regulation of lipid raft microdomains. Biochem Biophys Res Commun.

[23] Ho SH, Martin F, Higginbottom A, Partridge LJ, Parthasarathy V, Moseley GW, Lopez P, Cheng-Mayer C, Monk PN (2006). Recombinant extracellular domains of tetraspanin proteins are potent inhibitors of the infection of macrophages by human immunodeficiency virus type 1. J Virol.

[24] Markovic-Mueller S, Stuttfeld E, Asthana M, Weinert T, Bliven S, Goldie KN, Kisko K, Capitani G, Ballmer-Hofer K (2017). Structure of the full-length VEGFR-1 extracellular domain in complex with VEGF-A. Structure.

[25] Miranti CK (2009). Controlling cell surface dynamics and signaling: how CD82/KAI1 suppresses metastasis. Cell Signal.

[26] Liu WM, Zhang F, Moshiach S, Zhou B, Huang C, Srinivasan K, Khurana S, Zheng Y, Lahti JM, Zhang XA (2012). Tetraspanin CD82 inhibits protrusion and retraction in cell movement by attenuating the plasma membrane-dependent actin organization. PLoS ONE.

[27] Wei Q, Zhang F, Richardson MM, Roy NH, Rodgers W, Liu Y, Zhao W, Fu C, Ding Y, Huang C (2014). CD82 restrains pathological angiogenesis by altering lipid raft clustering and CD44 trafficking in endothelial cells. Circulation.

[28] Schulz D, Severin Y, Zanotelli VRT, Bodenmiller B (2019). In-depth characterization of monocyte-derived macrophages using a mass cytometry-based phagocytosis assay. Sci Rep.

[29] Chamberlain LH, Shipston MJ (2015). The physiology of protein S-acylation. Physiol Rev.

[30] Murphy J, Kolandaivelu S (2016). Palmitoylation of progressive rod-cone degeneration (PRCD) regulates protein stability and localization. J Biol Chem.

[31] Valdez-Taubas J, Pelham H (2005). Swf1-dependent palmitoylation of the SNARE Tlg1 prevents its ubiquitination and degradation. EMBO J.

[32] Lapalombella R, Yeh YY, Wang L, Ramanunni A, Rafiq S, Jha S, Staubli J, Lucas DM, Mani R, Herman SE (2012). Tetraspanin CD37 directly mediates transduction of survival and apoptotic signals. Cancer Cell.

[33] Jee B, Jin K, Hahn JH, Song HG, Lee H (2003). Metastasis-suppressor KAl1/CD82 induces homotypic aggregation of human prostate cancer cells through Src-dependent pathway. Exp Mol Med.

[34] Andreu Z, Yanez-Mo M (2014). Tetraspanins in extracellular vesicle formation and function. Front Immunol.

[35] Wu Q, Zhou L, Lv D, Zhu X, Tang H (2019). Exosome-mediated communication in the tumor microenvironment contributes to hepatocellular carcinoma development and progression. J Hematol Oncol.

[36] Sun W, Wang X, Li J, You C, Lu P, Feng H, Kong Y, Zhang H, Liu Y, Jiao R (2018). MicroRNA-181a promotes angiogenesis in colorectal cancer by targeting SRCIN1 to promote the SRC/VEGF signaling pathway. Cell Death Dis.

[37] Jiang M, Zhang W, Zhang R, Liu P, Ye Y, Yu W, Guo X, Yu J (2020). Cancer exosome-derived miR-9 and miR-181a promote the development of early-stage MDSCs via interfering with SOCS3 and PIAS3 respectively in breast cancer. Oncogene.

[38] Lo HC, Hsu JH, Lai LC, Tsai MH, Chuang EY (2020). MicroRNA-107 enhances radiosensitivity by suppressing granulin in PC-3 prostate cancer cells. Sci Rep.

[39] Kim K, Kim IK, Yang JM, Lee E, Koh BI, Song S, Park J, Lee S, Choi C, Kim JW (2016). SoxF transcription factors are positive feedback regulators of VEGF signaling. Circ Res.

[40] Park JJ, Jin YB, Lee YJ, Lee JS, Lee YS, Ko YG, Lee M (2012). KAI1 suppresses HIF-1alpha and VEGF expression by blocking CDCP1-enhanced Src activation in prostate cancer. BMC Cancer.

[41] Zhu J, Liang C, Hua Y, Miao C, Zhang J, Xu A, Zhao K, Liu S, Tian Y, Dong H (2017). The metastasis suppressor CD82/KAI1 regulates cell migration and invasion via inhibiting TGF-beta 1/Smad signaling in renal cell carcinoma. Oncotarget.

[42] Mamer SB, Chen S, Weddell JC, Palasz A, Wittenkeller A, Kumar M, Imoukhuede PI (2017). Discovery of high-affinity PDGF-VEGFR interactions: redefining RTK dynamics. Sci Rep.

[43] Ferrara N, Gerber HP, LeCouter J (2003). The biology of VEGF and its receptors. Nat Med.

[44] Pulkkinen HH, Kiema M, Lappalainen JP, Toropainen A, Beter M, Tirronen A, Holappa L, Niskanen H, Kaikkonen MU, Yla-Herttuala S (2021). BMP6/TAZ-Hippo signaling modulates angiogenesis and endothelial cell response to VEGF. Angiogenesis.

[45] Chai J, Du L, Ju J, Ma C, Shen Z, Yang X, Liang L, Ni Q, Sun M (2017). Overexpression of KAI1/CD82 suppresses in vitro cell growth, migration, invasion and xenograft growth in oral cancer. Mol Med Rep.

