# Supplementary material for: KAI1(CD82) is a key molecule to control angiogenesis and switch angiogenic milieu to quiescent state
Source: J Hematol Oncol. 2021 Sep 16;14:148. doi: 10.1186/s13045-021-01147-6 (PMC8444549; doi:10.1186/s13045-021-01147-6)

Supplemental Figure 1

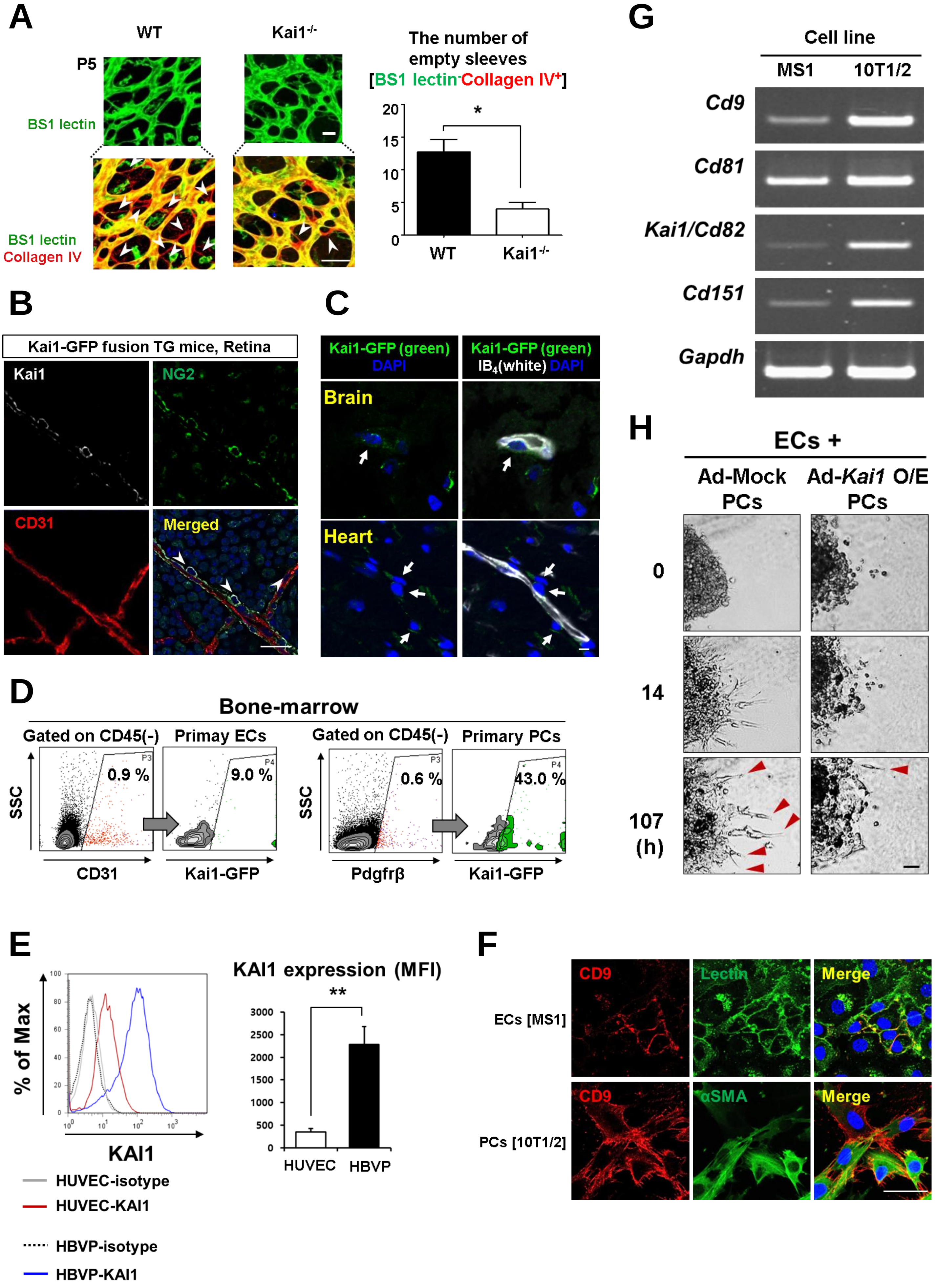

Supplemental Figure 2

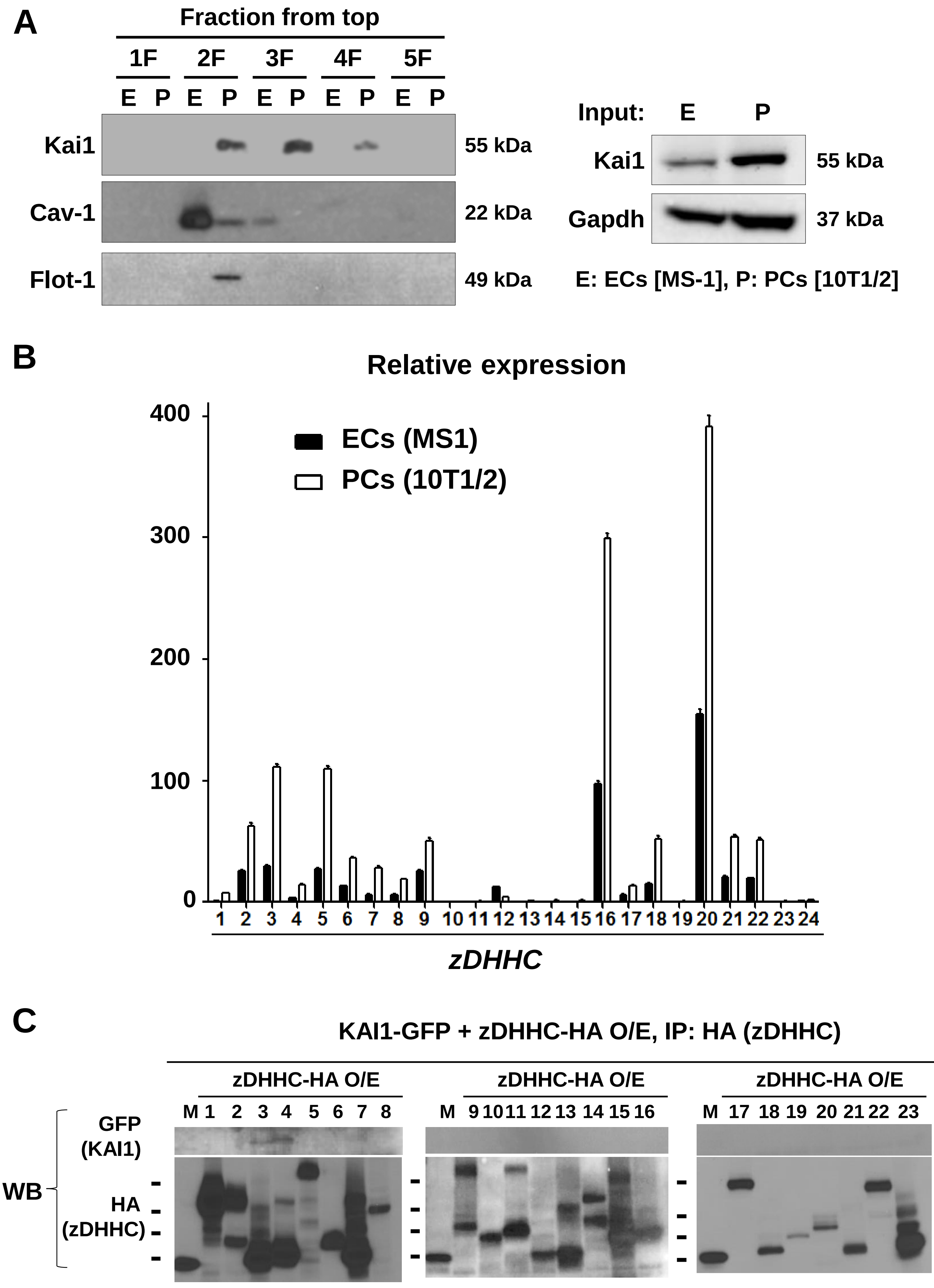

Supplemental Figure 3

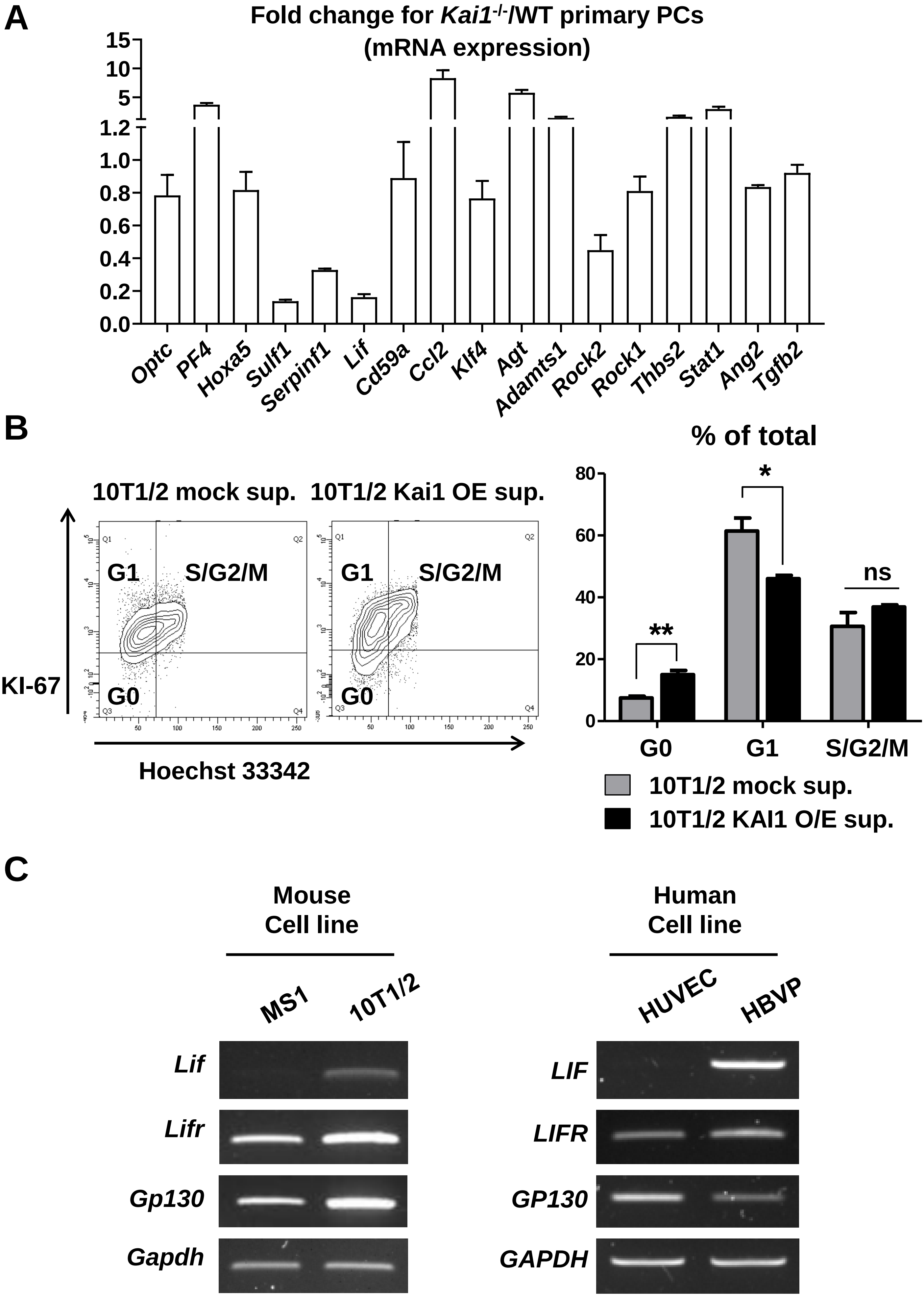

Supplemental Figure 4

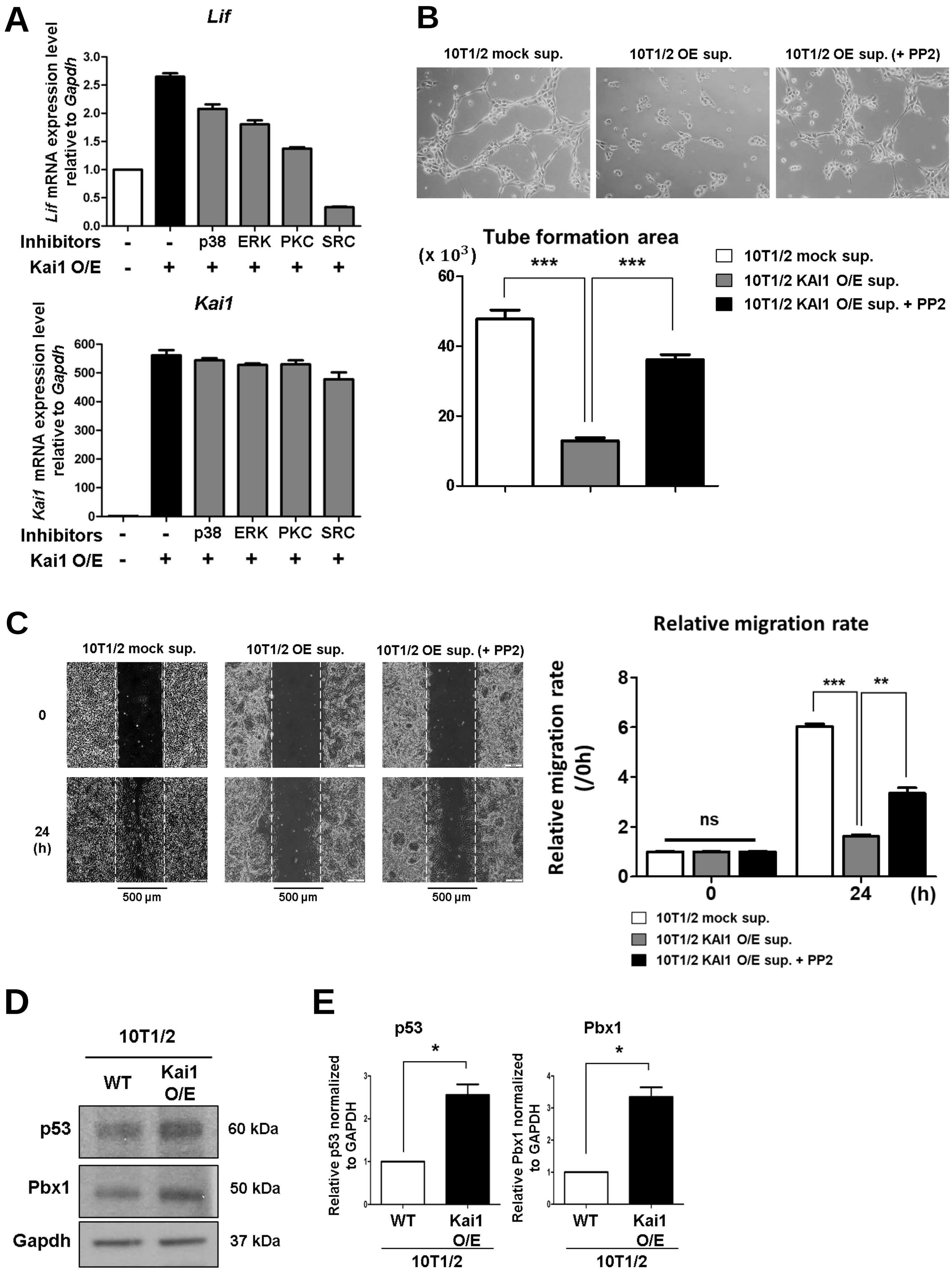

Supplemental Figure 5

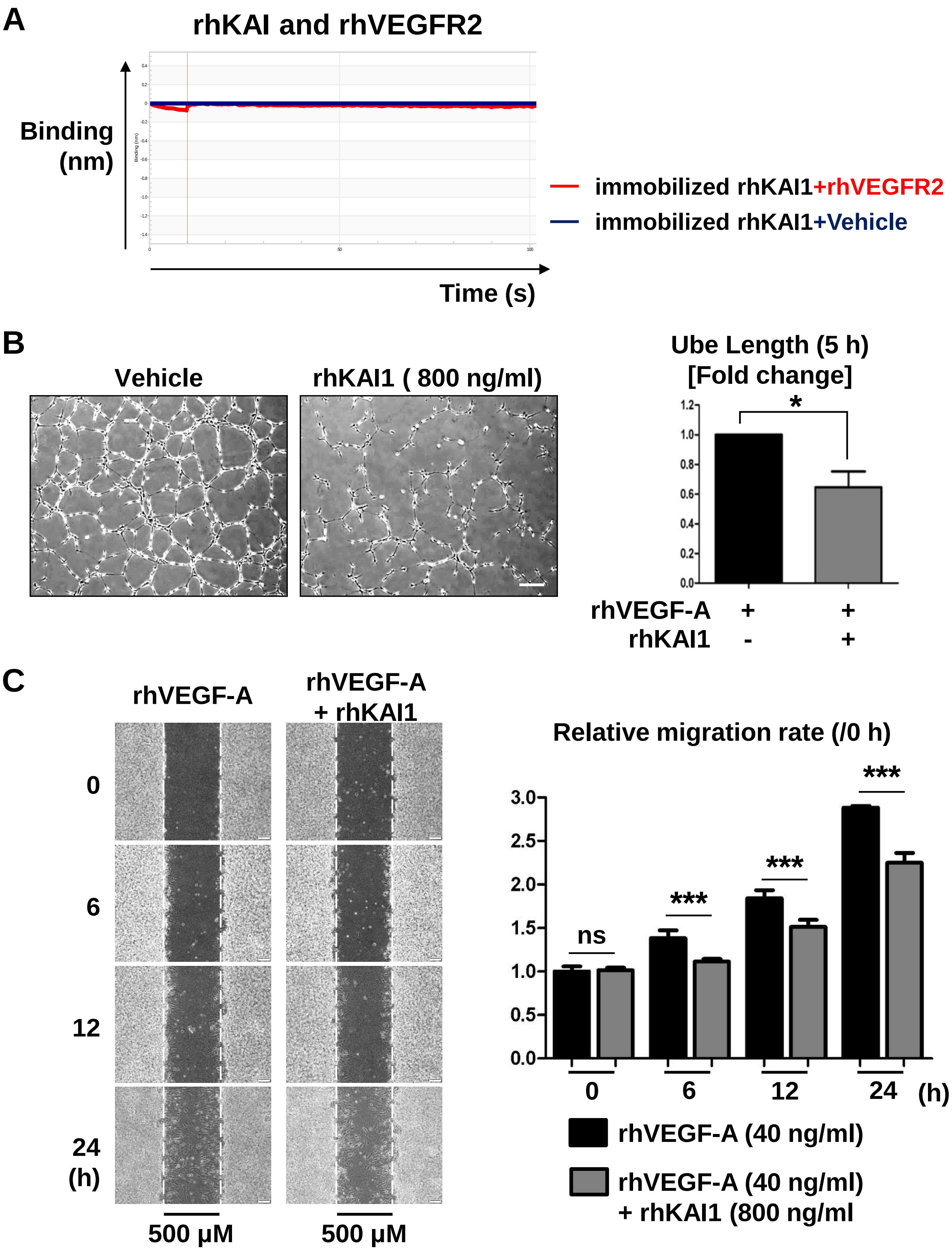

Supplemental Figure 6

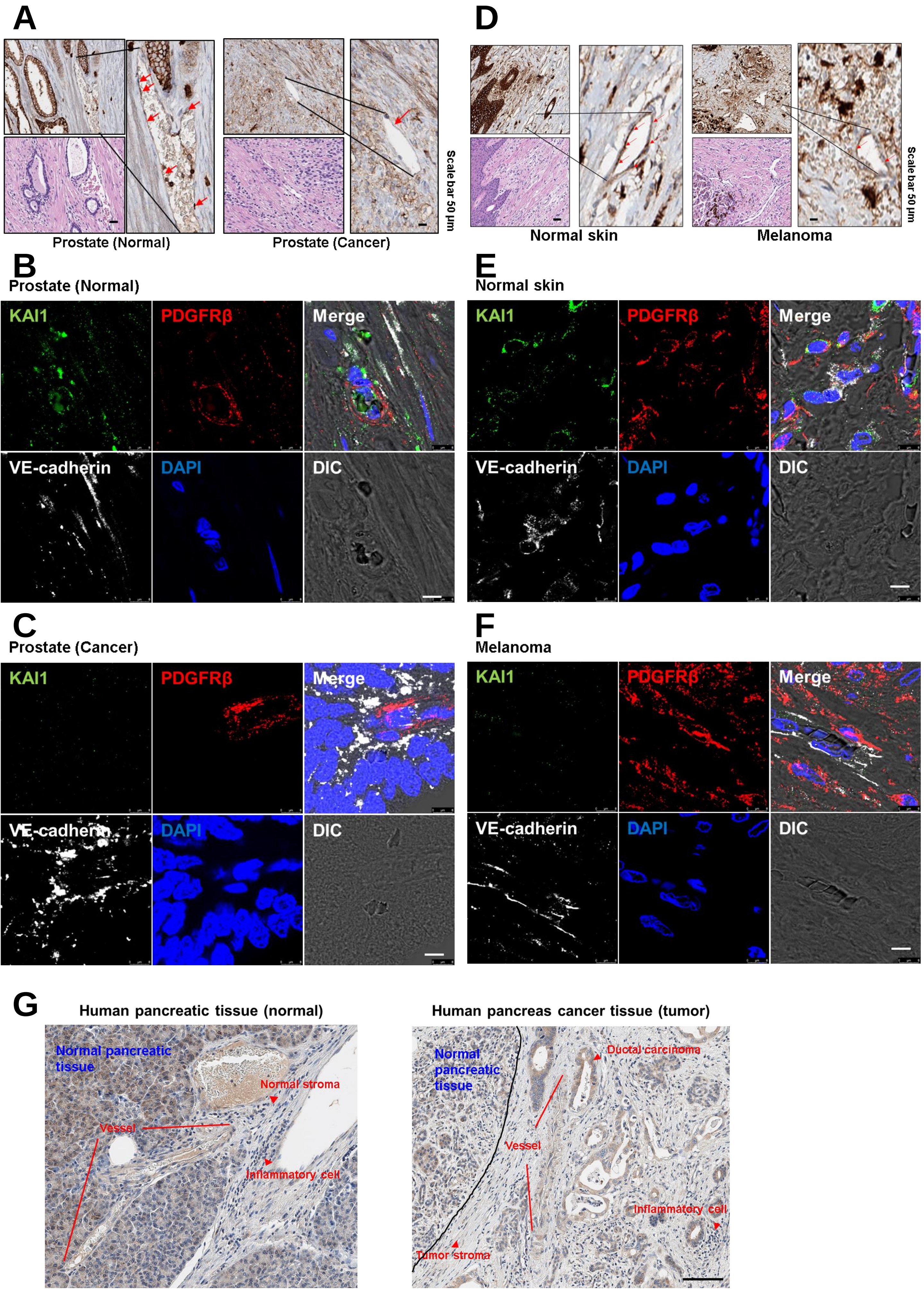

Supplemental Figure 7

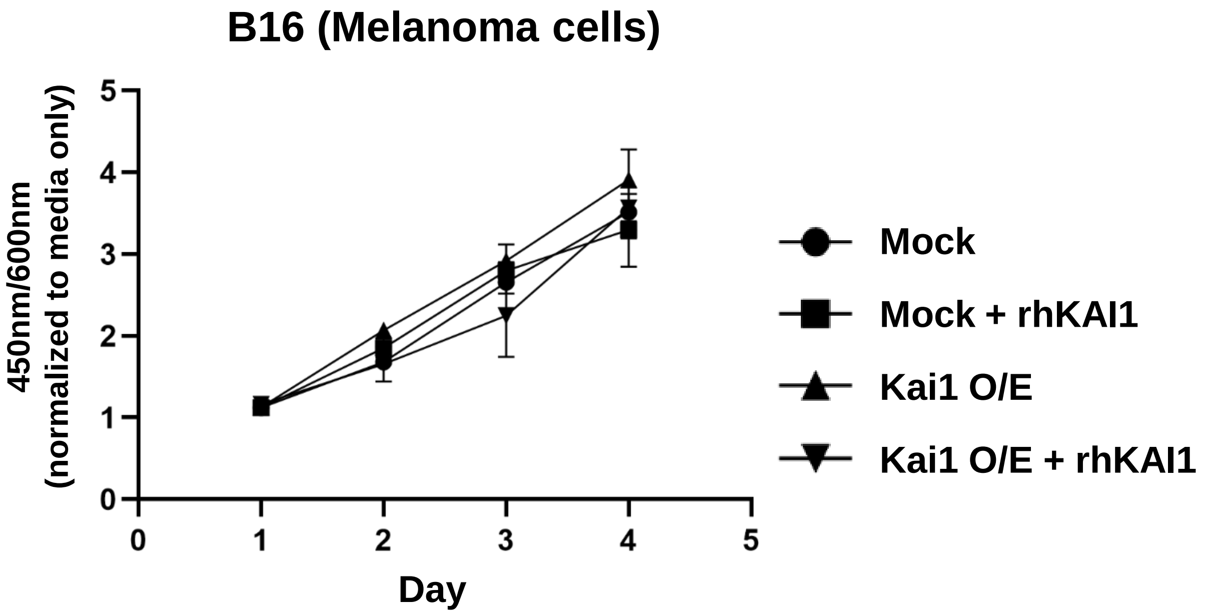

Supplemental Figure 8

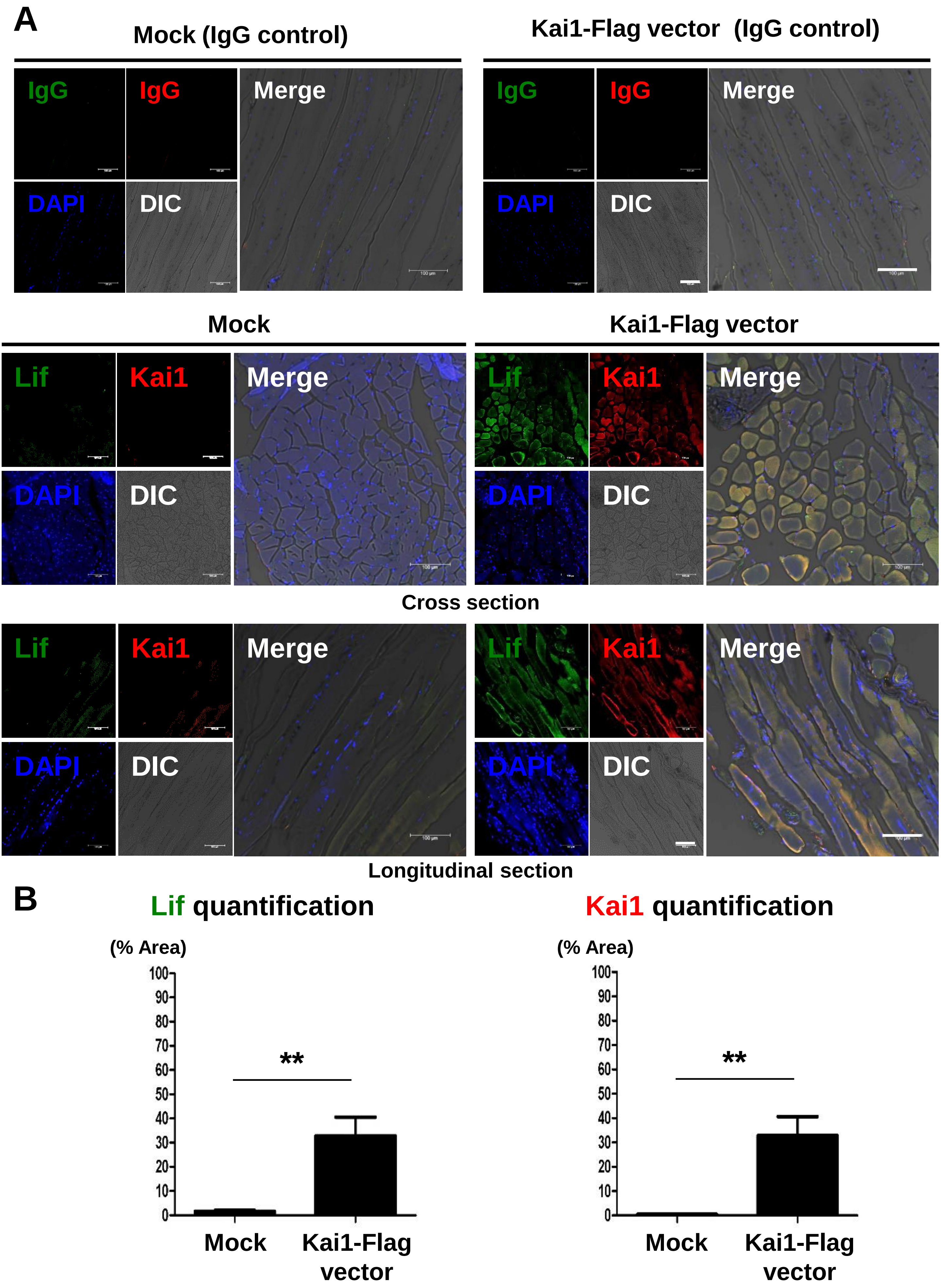

Supplemental Figure 9

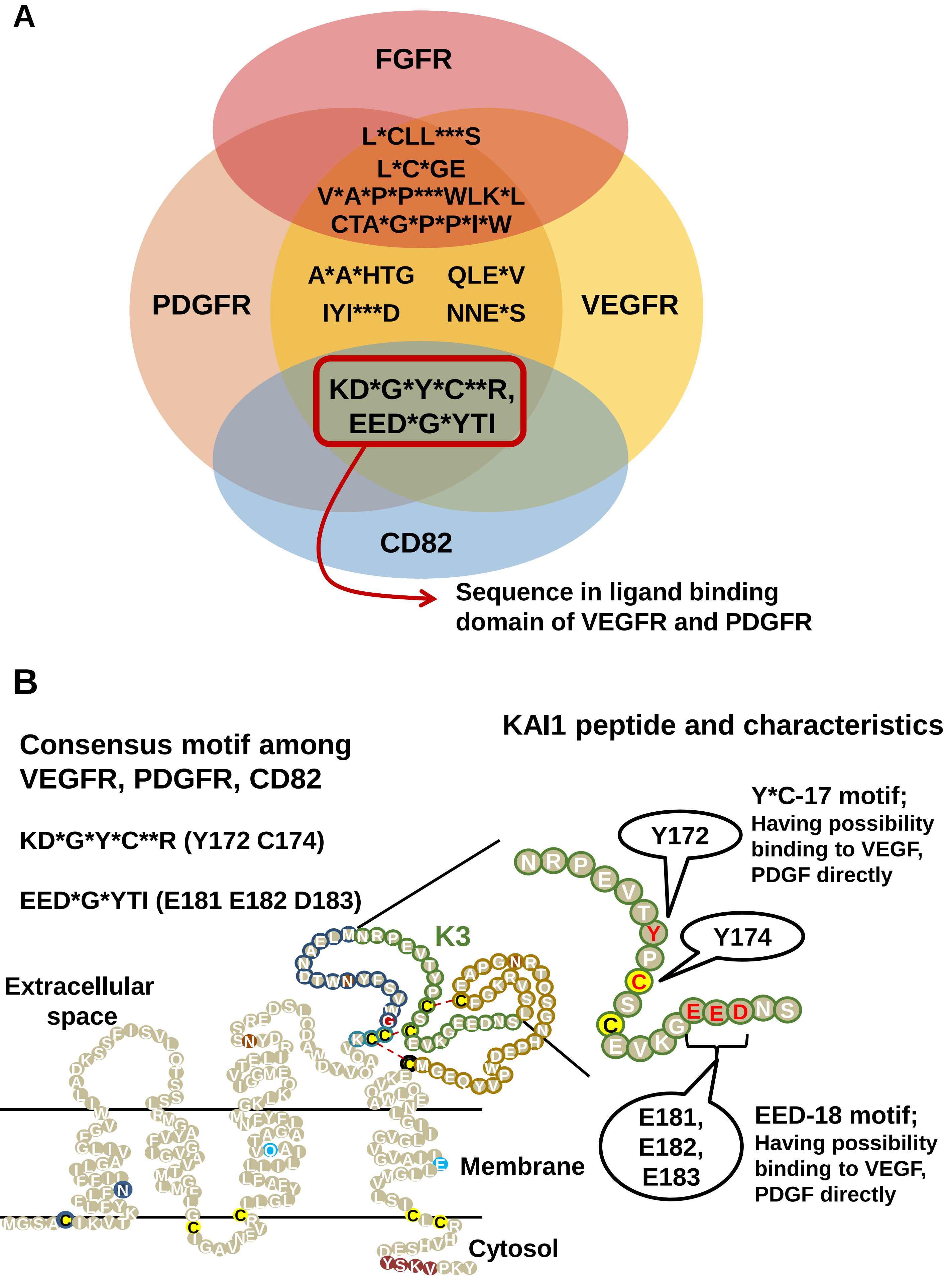

Supplemental Figure 10

KAI1 mutant peptide – VEGF binding test

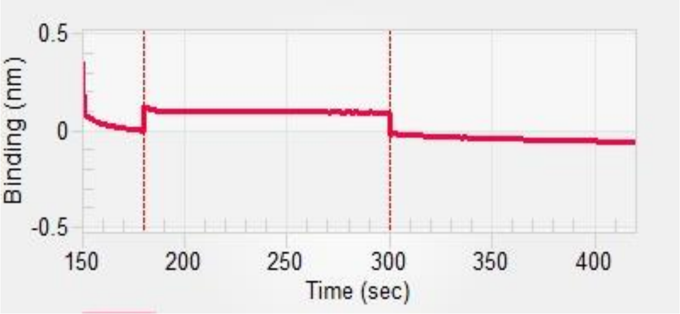

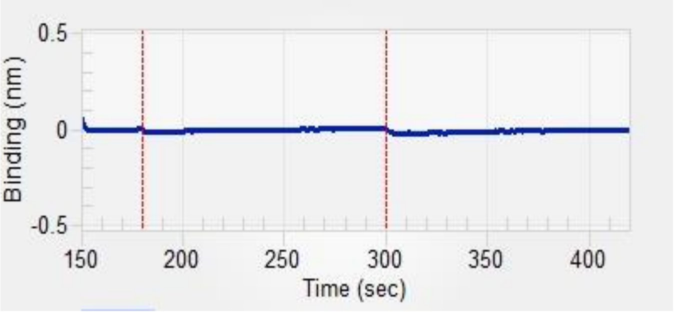

KAI1 WT peptide – VEGF

KAI1 mutant peptide - VEGF

KAI1 mutant peptide – PDGF binding test

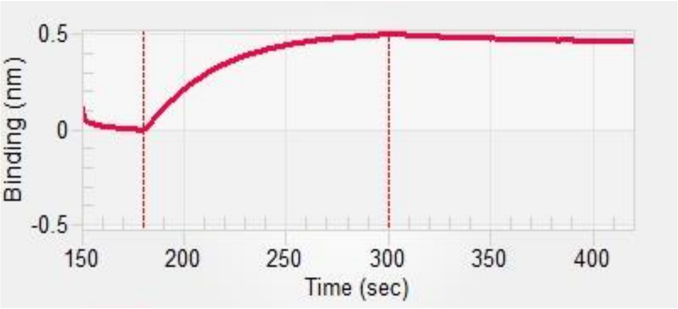

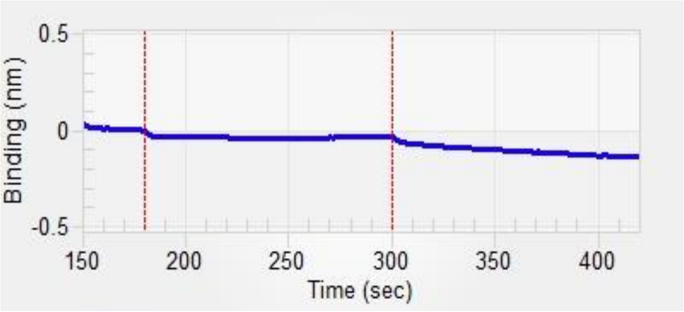

KAI1 WT peptide – PDGF

KAI1 mutant peptide - PDGF

Supplemental Figure 11

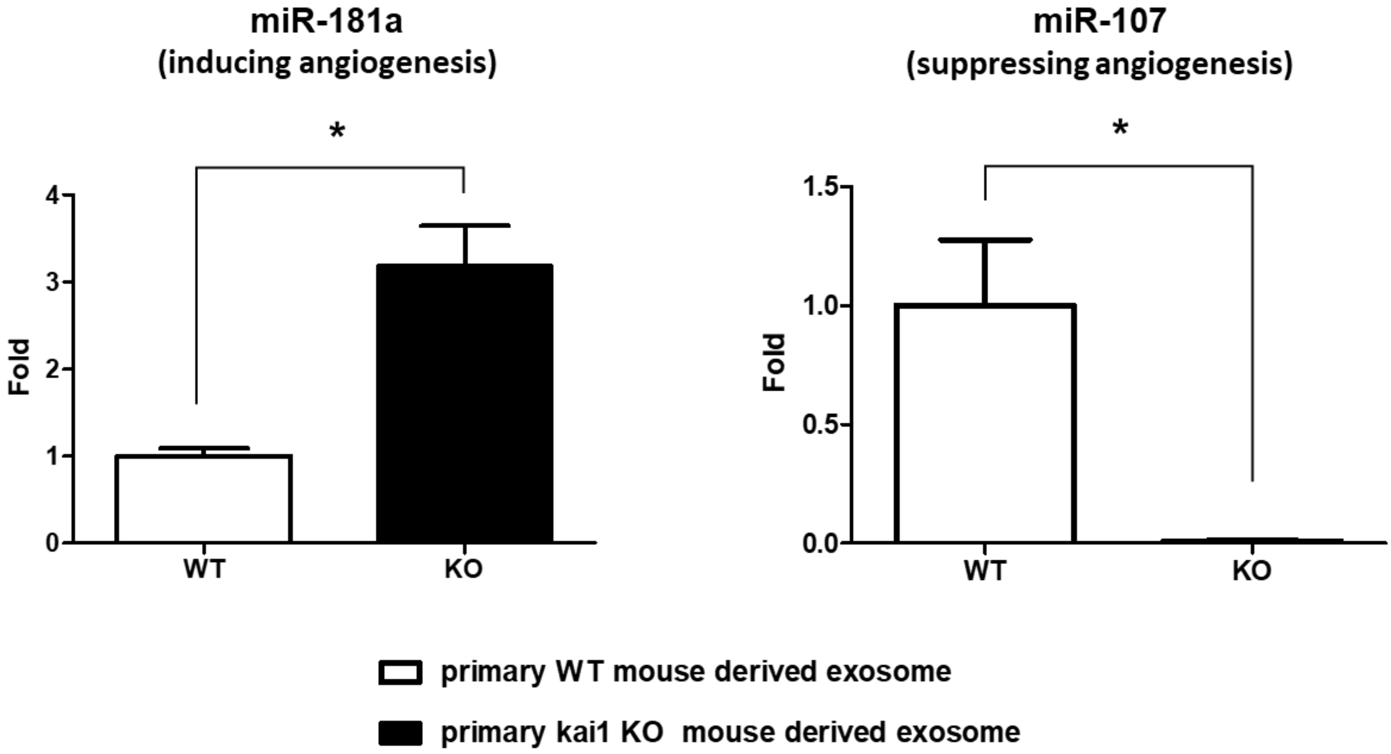

Video 1

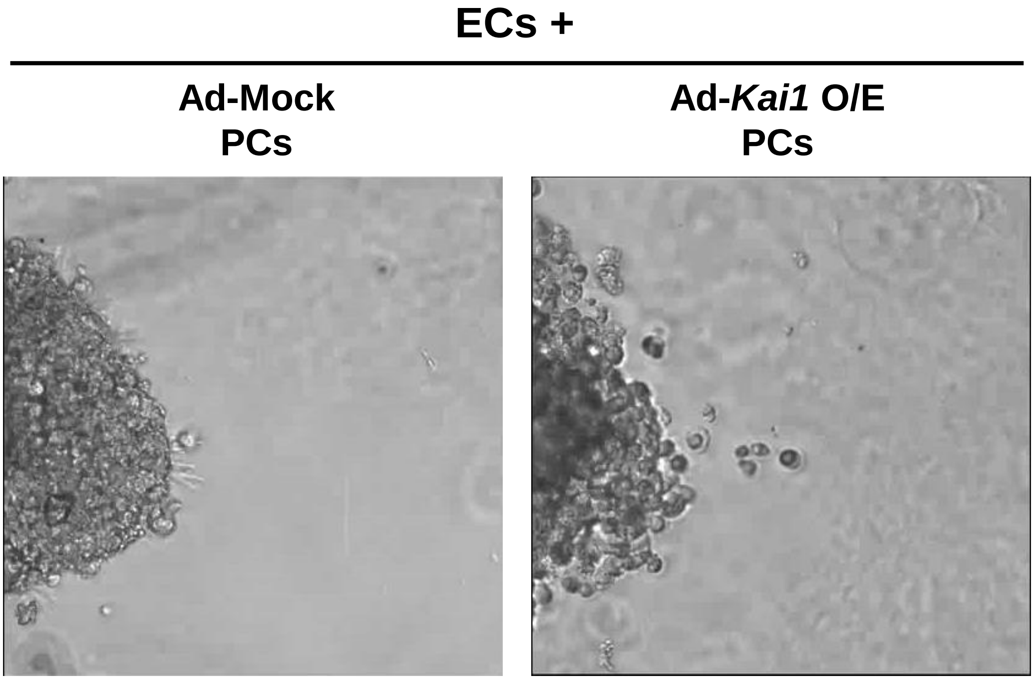

Supplement: Supplementary file 1 — Additional file 1: Figure EV1. KAI1 was expressed in PC more than EC and plays a role in the anti-angiogenic effect in PC. (A) Left, vessel regression (empty sleeves, BS-1 lectin-negative, collagen IV-positive) in the WT and Kai1−/− mice. Arrowheads, empty sleeves. Scale bar, 50 μm. Right, quantification of the obtained results (Means ± SEM, 4 technical replicates). (B) Confocal imaging in retinal of WT mice using Kai1 (white) antibody. Scale bar, 20 μm. (C) Confocal imaging of Kai1-GFP expression in brain and heart of the Kai1-GFP fusion transgenic mice. Scale bar, 5 μm. (D) FACS plot showing primary ECs (CD45−/CD31+) and primary PCs (CD45−/Pdgfrβ+) in the bone marrow of Kai1-GFP fusion transgenic mice. Left, KAI1 surface expression of human cell lines, HUVECs and HBVP, analyzed with FACS. Right, quantification of the obtained results. MFI (**p < 0.05, mean fluorescence intensity). (F) CD9 expression as an example in MS1 and 10T1/2 cells. Scale bar, 50 μm. (G) Expression of tetraspanins (Cd9, Cd81, Cd151) in ECs (MS1) and PCs (10T1/2) by using quantitative RT-PCR. (H) In vitro matrigel tube formation assay, performed using co-culture spheroids, consisting of ECs (MS1) and PCs (10T1/2), transduced with the indicated adenovirus at 0, 14, and 107 h, and observed using time-lapse microscopy. Scale bar, 50 μm. Figure EV2. KAI1 localizes at the lipid rafts of PCs and binds with zDHHC3 and zDHHC4. (A) Lipid rafts isolated from ECs (MS1) and PCs (10T1/2) using OptiPrep medium (frozen and crosslinks method). Endogenous Kai1 localization is confirmed. Flotillin-1 (Flot-1) and caveolin-1 (Cav-1) was detected lipid raft membrane fraction of MS1 and 10T1/2, respectively. Fraction 1; no cellular protein, Fraction 2; Lipid raft proteins. Fraction 3; Non-raft membrane proteins. Fraction 4 and 5; Cytosolic proteins. (B) Semi-quantitative RT-PCR analysis for zDHHC family member expression in MS1 and 10T1/2 cells. The genes were normalized by Gapdh (Means ± SEM, 3 technical replicate [file 13045_2021_1147_MOESM1_ESM.pdf]
